# Design, Challenges and Developments for 5G Massive MIMO Antenna Systems at Sub 6-GHz Band: A Review

**DOI:** 10.3390/nano13030520

**Published:** 2023-01-28

**Authors:** Sura Khalil Ibrahim, Mandeep Jit Singh, Samir Salem Al-Bawri, Husam Hamid Ibrahim, Mohammad Tariqul Islam, Md. Shabiul Islam, Ahmed Alzamil, Wazie M. Abdulkawi

**Affiliations:** 1 Department of Electrical, Electronic and Systems Engineering, Faculty of Engineering and Built Environment, Universiti Kebangsaan Malaysia, UKM, Bangi 43600, Selangor, Malaysia; 2 Space Science Center, Institute of Climate Change, Universiti Kebangsaan Malaysia (UKM), Bangi 43600, Selangor, Malaysia; 3 Department of Electronics & Communication Engineering, Faculty of Engineering & Petroleum, Hadhramout University, Al-Mukalla 50512, Hadhramout, Yemen; 4 Faculty of Engineering, Multimedia University, Persiaran Multimedia, Cyberjaya 63100, Selangor, Malaysia; 5 Electrical Engineering Department, College of Engineering, University of Ha’il, Ha’il 81481, Saudi Arabia; 6 Department of Electrical Engineering, College of Engineering in Wadi Addawasir, Prince Sattam bin Abdulaziz University, Al-Kharj 11991, Saudi Arabia

**Keywords:** 5G systems, massive MIMO, base station, smartphone, 5G antennas, 5G applications, sub-6 GHz

## Abstract

Massive multiple-input multiple-output (mMIMO) is a wireless access technique that has been studied and investigated in response to the worldwide bandwidth demand in the wireless communication sector (MIMO). Massive MIMO, which brings together antennas at the transmitter and receiver to deliver excellent spectral and energy efficiency with comparatively simple processing, is one of the main enabling technologies for the upcoming generation of networks. To actualize diverse applications of the intelligent sensing system, it is essential for the successful deployment of 5G—and beyond—networks to gain a better understanding of the massive MIMO system and address its underlying problems. The recent huge MIMO systems are highlighted in this paper’s thorough analysis of the essential enabling technologies needed for sub-6 GHz 5G networks. This article covers most of the critical issues with mMIMO antenna systems including pilot realized gain, isolation, ECC, efficiency, and bandwidth. In this study, two types of massive 5G MIMO antennas are presented. These types are used depending on the applications at sub-6 GHz bands. The first type of massive MIMO antennas is designed for base station applications, whereas the most recent structures of 5G base station antennas that support massive MIMO are introduced. The second type is constructed for smartphone applications, where several compact antennas designed in literature that can support massive MIMO technology are studied and summarized. As a result, mMIMO antennas are considered as good candidates for 5G systems.

## 1. Introduction

As the need for faster data speeds grows every day, Fifth Generation (5G) is set to become the cutting edge of wireless communications. The data rate (as high as 20 Gbit/s) and capacity, high reliability, low latency (1 ms), enhanced device-to-device communication, and increased flexibility are the primary objectives of 5G communication systems [1].

Massive multiple-input multiple-output (mMIMO), a key-enabling technology for next-generation and beyond networks, was created as a reaction to the global bandwidth bottleneck in the wireless communications sector. In order to offer power efficiency and a great spectrum with fairly simple processing, mMIMO uses arrays of antennas at both the receiver and transmitter [2].

**Figure 1:**
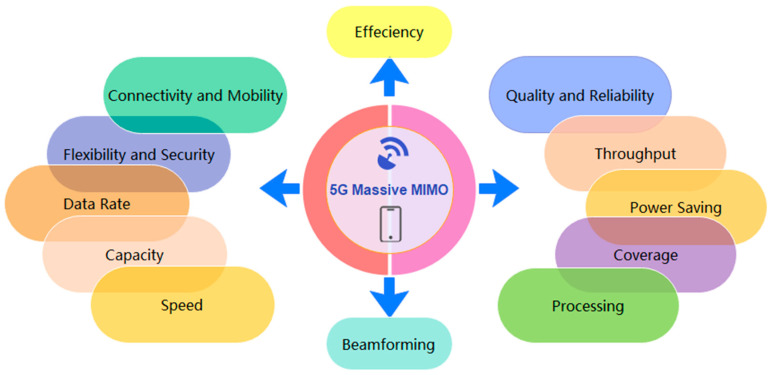
5G requirements and benefits of mMIMO.

mMIMO technology has achieved a major breakthrough in meeting the demand for high-quality mobile communication services that provide better coverage with lower power consumption without additional radio resources, reducing the bandwidth and transmission power. It can remarkably increase data throughput without adding more [3]. Several reviews have summarized the 5G requirements above-mentioned as meeting the benefits and properties of mMIMO, which can be concluded as shown in Figure 1 [2,4,5].

A key component of the design of the 5G communication system is the massive MIMO antenna. Massive MIMO represents an evolution of the MIMO technology that increases spectral efficiency and throughput by employing a significant number of active communication antennas [5].

In order to design a 5G mMIMO system, the type of element (antenna) and the application for which it is used must be determined. Therefore, the design can be defined as:

5G mMIMO = 5G antennas + Application

5G antennas make it possible to have multiple-input and multiple-output (MIMO). They consist of a large number of antenna elements in order to send and receive a large amount of data simultaneously. 5G antennas for base stations and smartphone applications have to be able to cover many frequencies. In this way, it becomes possible to achieve faster download speeds. Furthermore, it creates more capacity and connectivity for different devices. More bandwidth makes more data able to get through. For base stations, mobile phones, and other mobile devices such as connected cars, health monitoring equipment, and even industrial equipment, these antennas are vital for beamforming, steering, and reception [1,6].

The fundamental elements of wireless communication networks are base station (BS) antennas. At least one directional RF antenna, which might send and receive radio signals, may be part of it. These antennas are BSs that are tower-mounted and give consumers cellular access. Multiband base station mMIMO antennas are frequently contained within a single radome. In this instance, each antenna has a distinct port that can be attached to many BSs or parts of a single BS [7]. This is the main communication point for a single user or multiuser devices. 5G base stations are equipped with beamforming mMIMO antennas. Furthermore, an array of antennas can simultaneously focus and direct multiple beams to different targets on the ground [3].

The base station antenna defines an antenna that is used to send out a signal to more receivers and is suitable for indoor and outdoor applications. This type of antenna serves in satellite, GPS, GSM, WIMAX, WAN, LAN communications, …etc., and can be used to both receive and transmit a signal [8,9,10,11,12]. Mobile antennas have multiple advantages and are best suited for moving devices, from which you need to be able to communicate while moving around. These will need MIMO antennas for high-level performance antennas located on the edges and corners of the phones that support beamforming. A typical smartphone has approximately six antennas for high and low frequencies. MIMO, on the other hand, is a must for 5G, which needs two for the lower band and one for the upper band, of which 4 × 2 (or 4T2R) is one common combination. This indicates that the majority of new 5G phones, if not all of them, feature four antennas for cellular coverage. These antennas almost definitely will include automatic antenna adjusting capabilities [13]. To avoid interference, each individual antenna in a mobile phone should be placed as far away from the other antennas as possible. On a small phone, this is quite difficult. Fortunately, the operating frequency is high, as are the wavelength and antenna length. Here, it increases the number of antennas that must be integrated into a phone, especially since the majority of these standards support large-scale multi (MIMO) antennas [14].

Deploying 5G networks below 6 GHz requires a wide range of up to 20 Gbps and higher data rates. The 5G bands released are 3.3–4.2 GHz and 4.4–5 GHz, increasing the bandwidth to 100 MHz. In addition, to cover large areas, the base station has a mMIMO or 3D configuration that covers both the azimuth and vertical planes. Both the smartphone and the base station must increase the number of antenna elements below 6 GHz. At the higher frequencies of 5G, mMIMO arrays with significantly more radiating antenna elements will significantly minimize the network congestion and increase its capacity and throughput [15]. ITU-R has designated the mid-band of the sub-6 GHz (FR1) spectrum for the deployment of 5G communications, which comprises LTE n71 (470–698 MHz), n81–n83, n91–n94 (698–960 MHz), n74–n76(1.427–1.518 GHz), n65, n66(2.11–2.2 GHz), n30, n40, n38, n41, n 90 (2.3–2.69 GHz), n 77, n78, n79 (3.3–5 GHz), and LTE42/43/46/47 (3.3–5.925 GHz). Most of the conventional cellular mobile communication traffic is expected to be carried by FR1 bands. The large-scale sub-6 GHz MIMO antenna array is anticipated to be deployed inside the radiation aperture of the original 3G, 2G, and 4G antennas [15,16]. In the specified use scenarios, the 5G specification enables a maximum downlink data rate that is twice as fast as the uplink data rate. 5G requires at least a 4 × 4 downlink MIMO and at least a 2 × 2 uplink MIMO is recommended in the current deployment phases below 2.6 GHz [17,18].

## 2. 5G Massive MIMO at Sub-6 GHz

High data speeds and minimal latency are anticipated from fifth-generation mobile networks (5G), which will also boost the power efficiency and spectrum of wireless communication systems. For use in 5G systems, many technologies are being investigated. The mMIMO system is one of the most significant technologies anticipated to guide the development of 5G. One of the technologies that will support fifth-generation (5G) wireless systems is mMIMO [19]. A massive MIMO system is defined as an arrangement of an MU-MIMO system in which a base station and a terminal dispose of many antenna elements. Simultaneous communication with multiple users using the same resource is possible with multiple antennas, which provide higher spectral and power efficiency. In the case of a base station with multiple active antennas, it will be possible to communicate with a terminal (UE) on the same time–frequency resource through spatial multiplexing [20]. Additionally, the beamforming enhancement technology developed for these mMIMO systems can be used to reduce the transmit energy required by upgrading the base station equipment with a huge number of antennas, instead of deploying a new base station site [19,21].

Massive MIMO in (sub-6 GHz) 5G NR networks is an advanced antenna technology that improves spectral efficiency, network capacity, coverage, and feasible data rates. Massive MIMO uses multi-antenna elements to support multiple users simultaneously [22,23]. The essential parameters of mMIMO antennas can be considered as frequency bands, gain, isolation, radiation pattern, polarization, and beamwidth. One possible way to increase throughput and data transfer rates in current and future generations of mobile and wireless devices is to extend the bandwidth, as a higher bandwidth provides a higher data rate [24].

Reducing the mutual coupling effects between antenna elements in large-scale arrays with limited space requires several techniques suitable for such a crowded capacity of antenna elements without increasing the space between antennas. The low mutual coupling will offer high isolation and low correlations for the system without limiting the system’s performance. Several decoupling techniques have been put forth such as inserting a metamaterial wall to create a spatial band-stop filter or a spatial polarization-rotated wall, or utilizing metal structures between the parts of the antenna to add an additional coupling path [25]. It is challenging to implement such decoupling structures in a large-scale MIMO antenna since they all need to be fairly substantial in size. For BSs and less than −10 dB or lower for smartphone applications, the mutual coupling regarding a mMIMO array should ideally be less than −25 dB or even lower. Using beamforming methods could boost the gain and efficiency of a mMIMO system. To enhance the overall system performance and capacity, beamforming can be defined as a signal processing approach that is utilized with multiple antenna arrays on the receiver side and/or transmitter side for sending or detecting multiple signals from multiple desired terminals at once [26].

**Figure 2:**
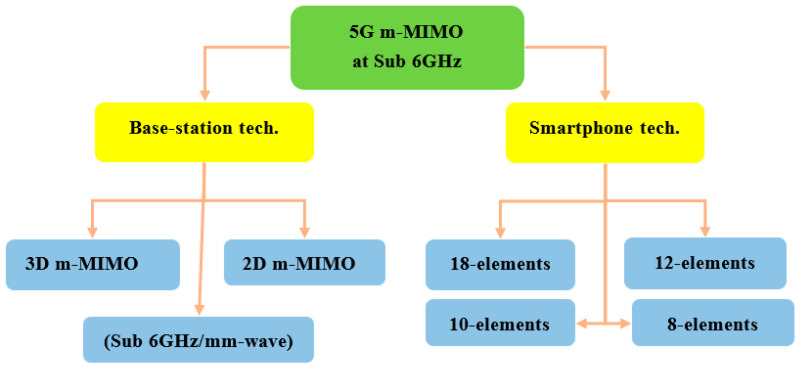
Techniques of applications for 5G massive MIMO at sub 6 GHz.

In order to investigate the types of applications, in this study, the types of 5G antenna can be divided into two kinds that support massive MIMO, base station, and smartphone antenna techniques. Figure 2 explains the application techniques used for 5G massive MIMO at sub-6 GHz.

### 2.1. 5G Massive MIMO Antennas for BS Applications

Many techniques are used in the architecture and configuration of antennas to design mMIMO systems for 5G sub-6 GHz applications. Many base station antenna designs attempt to cover dual band/multiband in compact volume and support mMIMO systems in base station applications.

#### 2.1.1. 2D Massive MIMO

**Figure 3:**
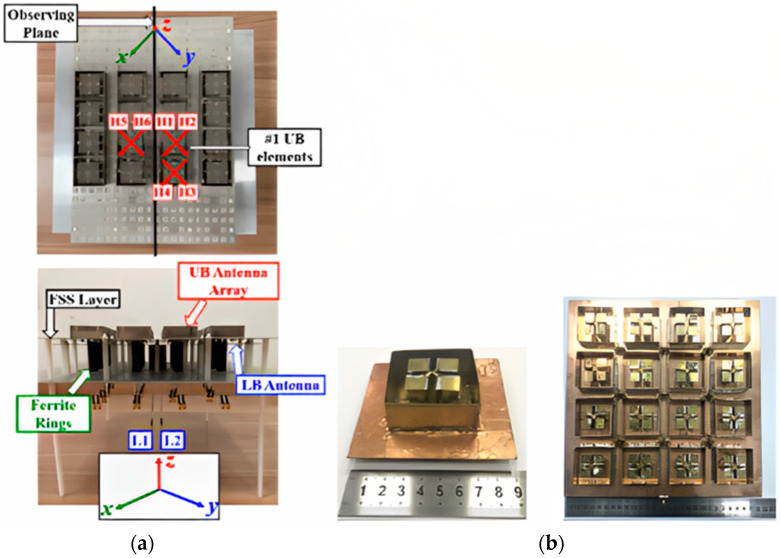
The fabricated prototypes: (**a**) (4 × 4) mMIMO of dual-band, dual-polarized shared aperture rectangular antenna array, top view; with side view, (**b**) (4 × 4) transformable dual polarized ME-dipole antenna for 5G array/mMIMO of dual-polarized differential feeding lines [30].

A rectangular planar lattice array technology is the most straightforward planar mMIMO configuration. For instance, an array of N×M is an arrangement of the planar array [27]. The 2D model element is one of the most popular used for the design of mMIMO. For use in 5G base stations, dual-band antenna arrays with dual polarization and common aperture have been introduced. The created antenna array includes a (4 × 4) planar MIMO array that operates at the 3.3–5 GHz band (upper band-UB), in addition to one antenna element that works at the 0.69–0.96 GHz band (lower band-LB). The UB antenna array can be defined as a large rectangular grid array that has 16 antenna elements that are designed for use in practical applications of MIMO. Figure 3a illustrates how in-band and cross-band mutual coupling amongst the UB and LB antennas are suppressed using three decoupling methods: a ferrite chock ring, a rectangular ring resonator, and a unique baffle design. The UB and LB antenna arrays are efficiently integrated with a small size of (0.93 × 0.93 × 0.17) λL, employing decoupling technology. In HB and LB antennas, a dual-band antenna array achieves a bandwidth of 41% and 32.7%, respectively. High cross-band port isolation (more than 30 dB) is provided. With average gains of 7.3 dBi and 8.6 dBi, respectively, the UB and LB antennas also produce stable radiation patterns. All operational ranges have better than 90% radiation efficiency. Compact volumes were used as GSM antenna elements for the first time in large-scale MIMO antenna arrays up to 6 GHz with a shared aperture. Additionally, the antenna array’s overall height is 0.17 L, which is considerably shorter than the present dual-band BS antennas. For low-profile, wide-band common-aperture 5G mMIMO BS arrays, the decoupling technique thus offers an effective option [28,29].

Low cross-polarization (X pol), high gain, and isolation properties could be accomplished with the antenna by combining a dual-polarized differential feeding arrangement with a complementary magneto electric (M.E.) dipole antenna. A modified H-shaped (1–16) differential feed network was created to feed the 16-antenna array to utilize these array properties, as illustrated in Figure 3b. In this case, the vertical cross-sections with dotted slots are positioned between adjacent dipole units to further improve the array isolation. A system of high-capacity MIMO antennas could be created through the removal of an H-shaped differential power feeding network and the underlying substrate. Measurement findings show that an antenna element in the 5G frequency band can achieve a low X-pol of −35.7 dB and a high gain of over 8.1 dBi (i.e., 3.3 to 5.1 GHz). The antenna array can be used to create a 32-channel capacity MIMO antenna technology, thanks to its high gain of 17.3 dBi and low envelope correlation coefficient (ECC) value of 0.004 [30,31].

For 5G BS applications, dual polarization (D.P.) mMIMO (32T/32R) wideband tightly spaced big antenna arrays have a whole distinct structure and interconnect (mutual coupling) analysis. By aligning two chamfered aerial bowtie dipole antennas in the orthogonal directions and employing 2-coaxial cables for feeding, dual polarization is made possible. The model includes chamfering, which increases the port isolation to port, and 2-arms of the bowtie aerials. Uni-directional radiation is produced through the placement of a metal ground plate at a λ/4 distance. The radiating component’s expected operating range is between 3.5 and 4.0 GHz. Simulation tests show that the anticipated dual polarization 2-chamfered aerial bowtie dipole antenna has a SWR of 1.5 and bandwidths between 2.8 and 4.0 GHz, a −27 dB isolation between the two ports, a 9.1 dBi gain for both polarizations, and a mutual coupling of roughly −25 dB. The antenna features a simple, easily-manufacturable design with an overall height of 0.25λ_0_ [32,33].

Based on patch antennas, a mMIMO antenna system design is described. Each port in the array has a (2 × 2) patch antenna sub-array with a distinct phase excitation at every one of the elements to tilt the beam into a different direction and produce lower correlation coefficient values. The array has 16 ports (64 elements) in total. To deliver the beam-tilts, a fixed progressive phase feed network has been designed [34]. The antenna system uses a three-layer FR-4 substrate with a total size of 33.33 cm × 33.33 cm × 0.16 cm and is designed to operate at 3.6 GHz with a 230 MHz bandwidth, good isolation between the neighboring ports at a minimum of 25 dB, and achieved gains of 5.4 dB for each port [34].

**Figure 4:**
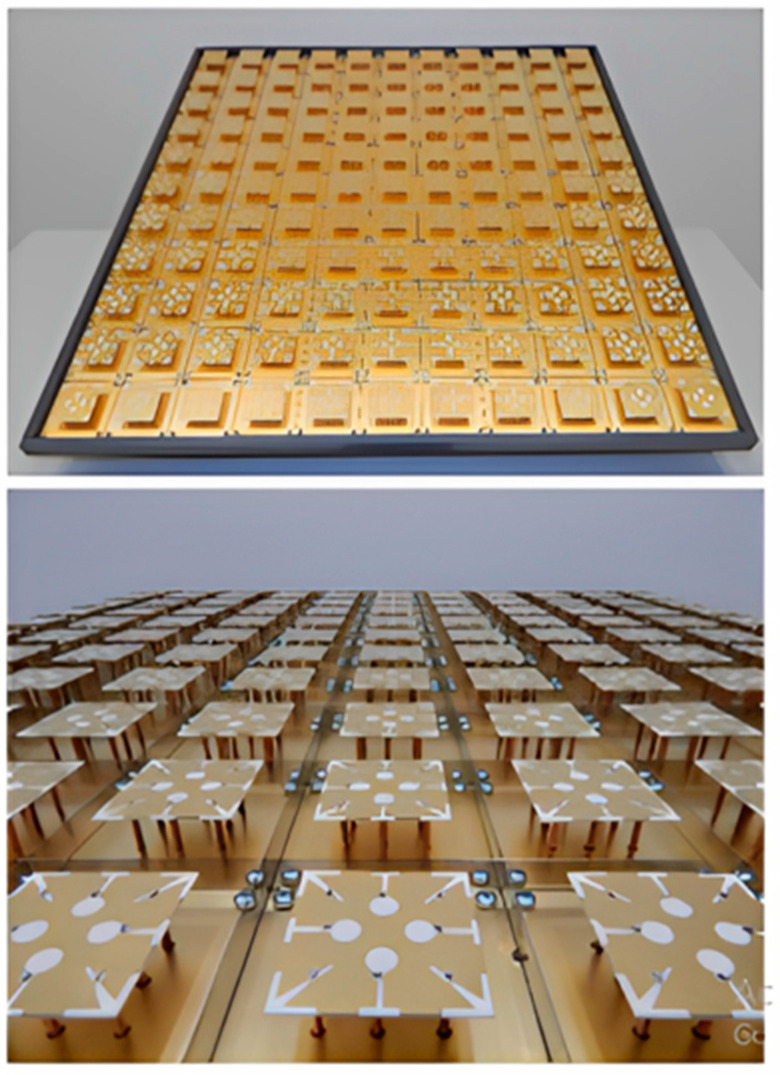
The prototype of an (11 × 11) mMIMO array of multimode elements has 484 antenna ports [35].

For a sizable indoor MIMO base station, a portable ultra-wideband MEA was investigated. The antenna’s design was based upon the simultaneous activation of several characteristic modes in every MEA element. Therefore, an (11 × 11) mMIMO array with a size of 70 cm by 70 cm and 121 physical antenna elements could be used to construct an effective 484-port antenna, as can be seen in Figure 4. Compared to the standard cross-dipole MEA, this resulted in a 54% size decrease. The antenna has a reflection coefficient of <−10, an intra-element and inter-element interconnection of ≤−20 at the antenna port, and operates over a very broad frequency range of 6 to 8.5 GHz. It uses the 3D radiation patterns to calculate the total antenna efficiency, which is around 70% for all four ports [35,36].

Wider carrier bandwidth, native mMIMO, and minimal latency are features of a sample 5G air interface. It has been shown that all physical channels could be designed to function with small beams. Eight streams of 64 QAM can be sent simultaneously for a single UE with eight antennas, and 5 Gbps throughput is possible at 200 MHz. It is possible to obtain a 10 Gbps throughput for multiple UE through evenly distributing such UEs in an outdoor setting. Additionally, this system was created using a commercial LTE hardware and software platform, and our top priorities regarding engineering were size, cost, and the power consumption limits. mMIMO may thus be a workable solution for the 5G wireless system. Future tests will examine increased multiple UE throughputs, multiple cell handover, velocity robustness, and collaboration [37].

As BS antennas consist of mMIMO systems, new broadband partial ground plane microstrip antennas and microstrip planar arrays are being developed. For upcoming 5G networks, broadband antennas and planar arrays are being designed for sub-6 GHz bands. Compared with the millimeter frequency band, this band has better coverage. Line-of-sight issues and losses due to atmospheric attenuation affect this mm frequency. The Roger 3003 substrate was used to build the antenna. The substrate was 50 mm × 50 mm × 1.5 mm in size, whereas 16.3 mm was the partial ground plane length, and 0.035 mm was the thickness of the PCB’s metal layer substrate. However, the size of the microstrip feed line had a length of 17 mm and width of 2.5 mm, respectively. The patch size was length 19.5 mm × width 17 mm, and this design had inset 1 and inset 2, which had the same dimensions (length 3 mm × width 0.5 mm) [38].

#### 2.1.2. 3D Massive MIMO

The varying channel characteristics (circular, planar, and cylindrical) resulting from various antenna array designs substantially impact the entire system’s performance. Generally, array dimension designs that are circular or planar show a considerable reduction, with the beam only being horizontally adjustable. These configurations also fall short of the escalating capacity demands. To address this shortage, it is suggested that 3D massive array configurations are adopted such as hexagons, cylinders, triangles, etc. [39].

**Figure 5:**
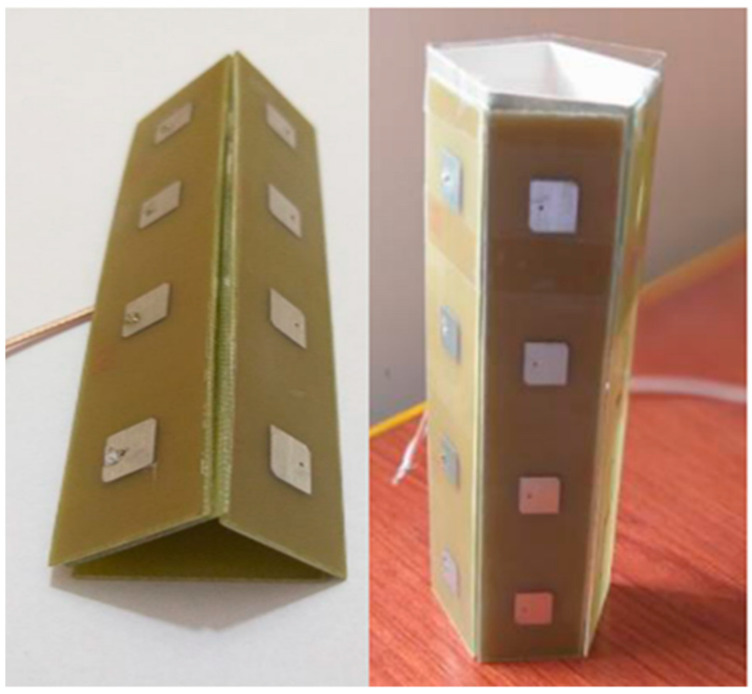
Manufactured prototype of the hexagonal (5 × 4) mMIMO of the patch antenna array [40].

Two types of 3D techniques are listed. The first type depends on the arrangements of elements in a three-dimensional array. A compact mMIMO antenna system with 1 × 4 (sector) sub-array configurations operating in the sub-6 GHz band for 5G base stations was designed and decomposed into various configurations from numerous array topologies, and cubic and stacked polyhedral arrays (rectangular, triangular, and hexagonal) for array design, as shown in Figure 5. A mMIMO process can enhance a system limit by more than ten times while increasing its energy efficiency by a hundred times. Each sector contains components for a 1 × 4 sub-array and can have up to five sectors. The single sector has three layers, with the top layer containing (1 × 4) patches, while the bottom and center layers contain a separate ground plane and are responsible for the organization. The complete system could operate in either a massive MIMO show mode with beam steering capability or a single port mode. The frame’s 140 MHz intentional data transmission spans the sub-6 GHz spectrum at frequencies between 3.36 GHz and 3.50 GHz. The size of a unit sub-array in terms of its length, width, and height was 280.5 mm × 56.1 mm × 2 mm, respectively. The gain of a single port was realized at 12.95 dBi, and a single panel with five sectors arranged in a rectangular configuration had a total addition of 19.73 dBi. All of the ports had a mutual coupling of about −16 dB. The operational frequency of the radio antenna array system was set between 3.3 GHz and 3.8 GHz. This is because the sub-6 GHz spectrum was set aside and concentrated internationally to support 5G [40].

To make it easier to analyze mutual coupling, the 24 antenna units were designated consecutively from 1 to 24 and arranged in three stacked stages with the letters A, B, and C. The mMIMO of the dual-polarized antenna array provides a maximum mutual coupling of <−35 dB between each pair of ports in this array. The created array was compensated by 18 low-profile sub-arrays. There were four single units in each sub-array. Every one of the antenna units comprised a single port that was vertically polarized, and a single horizontally polarized port connected to the power splitters served as the feeding network. For a dual port convertible patch antenna of the same size, a stacked patch design that incorporates a feed network offers higher gain and less mutual coupling. The three stacked stages of regular hexagonal walls in the array layout, which depend on the Turning Torso building model, had increasing twist angles of 20 degrees between the adjacent levels. Every stage had six sub-arrays. As a result, 18 sub-arrays were dispersed throughout an area with a 324 mm radius. This arrangement decreased the array’s radial size by increasing the longitudinal vertical stack size. The array’s overall volume was 648 mm by 648 mm by 258 mm and included 288 patches and 144 ports, three stacked stages, A, B, and C, with a total of 24 antenna units, which were successively designated (1–24) for the facilitation of a mutual coupling study that followed. The mMIMO of a dual-polarized antenna array decreased the maximum mutual coupling between every two array ports to <−35 dB [41].

**Figure 6:**
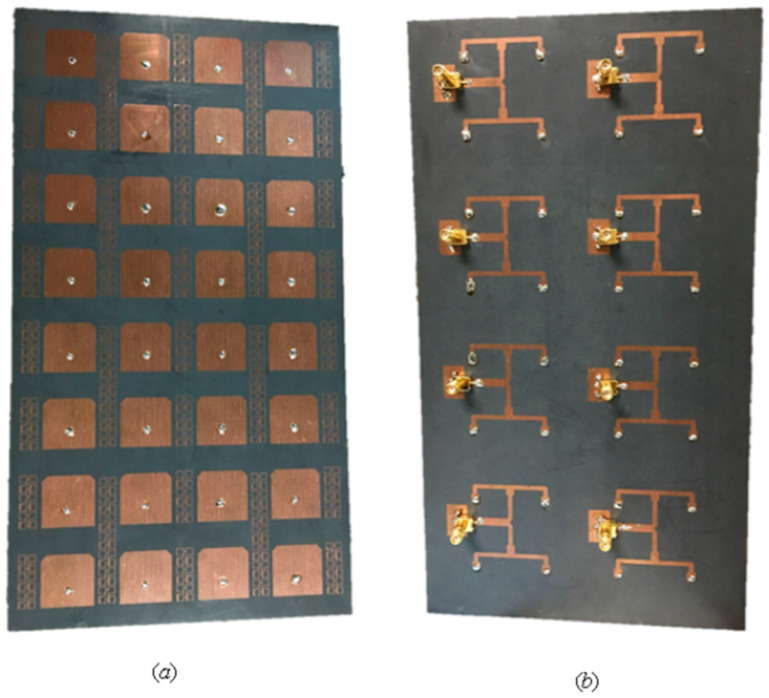
(**a**) Top view, (**b**) bottom view of fabricated single-side prototype [42].

For a future 5G base station with a measured bandwidth of 250 MHz, a relatively high isolation mMIMO antenna with 32 elements is investigated in [42]. This antenna can span the frequency range of 3400–3650 MHz. The epsilon negative and almost zero refractive index characteristics of the suggested design’s operational working principle are intended to concurrently enhance the isolation and overall performance of the MIMO antenna system. To back up this assertion, a specific ENG/NZRI/DNG metamaterial unit cell is also examined. The proposed MTM consists of four small, square-shaped splatted components as shown in Figure 6. The proposed MTM-based technique permits significant decoupling of up to 32 dB between the small array elements with ECC 0.0001 and the proposed radiating MIMO antenna elements, in contrast to conventional isolation solutions [42].

**Figure 7:**
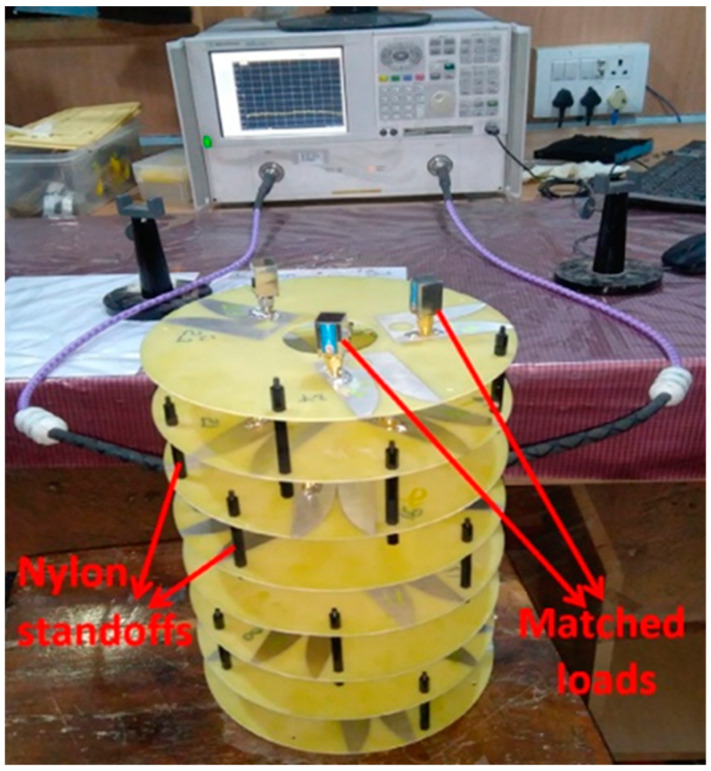
Fully-assembled 24-antennas of the MIMO array during testing with VNA for S-parameters [43].

A nature-inspired MIMO antenna configuration that can provide lower-bound wireless channels in the lower 5G band (3–4.2 GHz) has been achieved, as shown in Figure 7. In principle, the cross-correlation between antenna elements is decreased by applying the golden angle (137.5°) concept to the cylindrical configuration of tapered slot antenna arrays. The configuration of a cylindrical array offers high directivity and narrow beam pointing in all spatial directions. The golden angle helps in positioning end-fire radiating tapered slot antennas (TSAs) to prevent spatial overlapping of the radiated individual antenna element fields. TSA elements arranged in 24 cylinders were used to test this theory. The ECC value was <0.01 at 3 GHz–4.25 GHz. According to the simulations and measurements, the above frequency range exhibited extremely excellent impedance matching and mutual coupling between the antenna elements. It is thought that using the golden angle concept with MIMO antennas will enable it to use dense massive MIMO [43].

**Figure 8:**
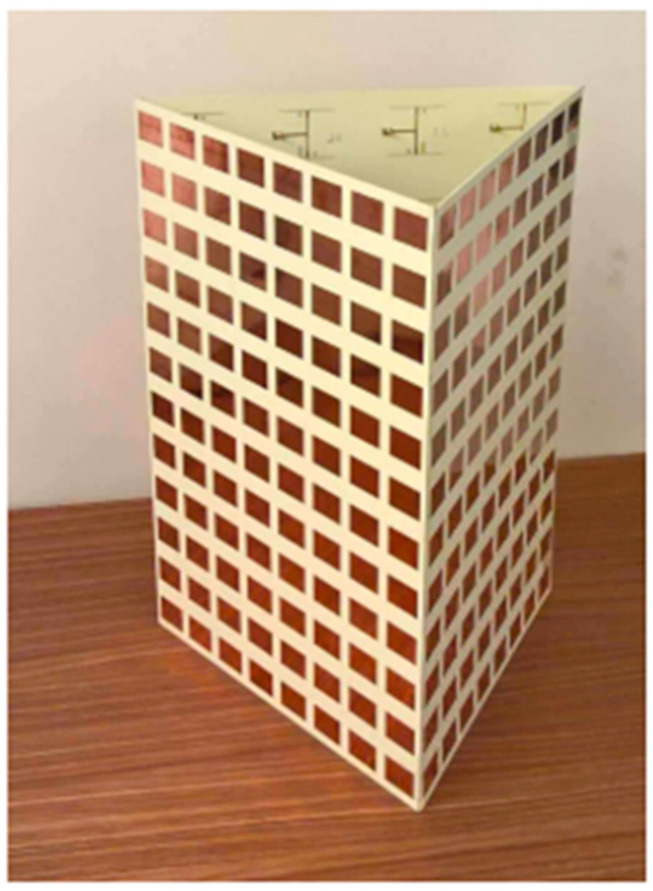
Triangular 72 port mMIMO system prototype [44].

A 72-port/288-antennas triangular-shaped massive multiple-input/multiple-output (mMIMO) antenna system was achieved for 5G base stations, as shown in Figure 8. Each side of the antenna system contains three layers and 24 ports with an overall size of 44.4 cm × 29.6 cm × 0.1524 cm. A single-port (sub-array) is composed of a ground plane in the middle, a main with pre-computed phases on the bottom, and a (2 × 2) patch on the top layer. Each sub-array (one port) is fed by tilting the beam direction relative to the other to obtain unrelated patterns. For the moment, the antenna system supports two operating modes. The operation uses multiple ports simultaneously (72-port MIMO) and a mMIMO array simultaneously (using beam-switching). All characteristics were measured, and the attained bandwidth was 100 MHz, which covered the band at 3.45–3.55 GHz. The gain of a single port was 9.41 dBi, the envelope correlation coefficient (ECC) was ≤0.1198, and the efficiency was 64%. Beam steering techniques were introduced and used to direct the beams on each side with 24 ports to various locations in space based on non-uniform port configurations, in contrast to most conventional methods that use identical array elements. The received 13-switchable beams had a center coverage angle of up to 34° at a gain and height of up to 19.5 dBi [44].

Other types of 3-dimensions depend on the full dimension of the beamforming pattern in (3D) space by using active antenna elements with a multi-beam system. With beamforming technology, a MIMO base station massive antenna focuses radio signals directly on the user and their device, as opposed to broadcasting them in all directions, which causes an increase in the efficiency as it reduces interference. Beamforming antenna arrays that improve the throughput of mMIMO systems reduce intra- and inter-cell interference. In a beamforming antenna array, the signal received by each antenna element is adaptively shaped to increase the wireless communication system’s overall efficiency and gain. Complex weights are multiplied when signals are detected from various BS antennas.

Active antenna systems (AAS) with two-dimensional (2D) planar array structures are used in FD-MIMO. This permits adaptive electron beamforming and fitting numerous antenna elements into a functional base station compact design in three-dimensions (3D) [45]. The array of active transceivers is integrated into an array of passive antenna elements using the advanced BS technology known as AAS. This technology enables adaptive control of the gain, beamwidth, and transmit beam tilt using active electronics that are directly connected to each element. To enable adaptive beamforming for elevation and traditional azimuth measurements, active and functional antenna elements must be positioned in both the vertical and horizontal orientations [27].

**Figure 9:**
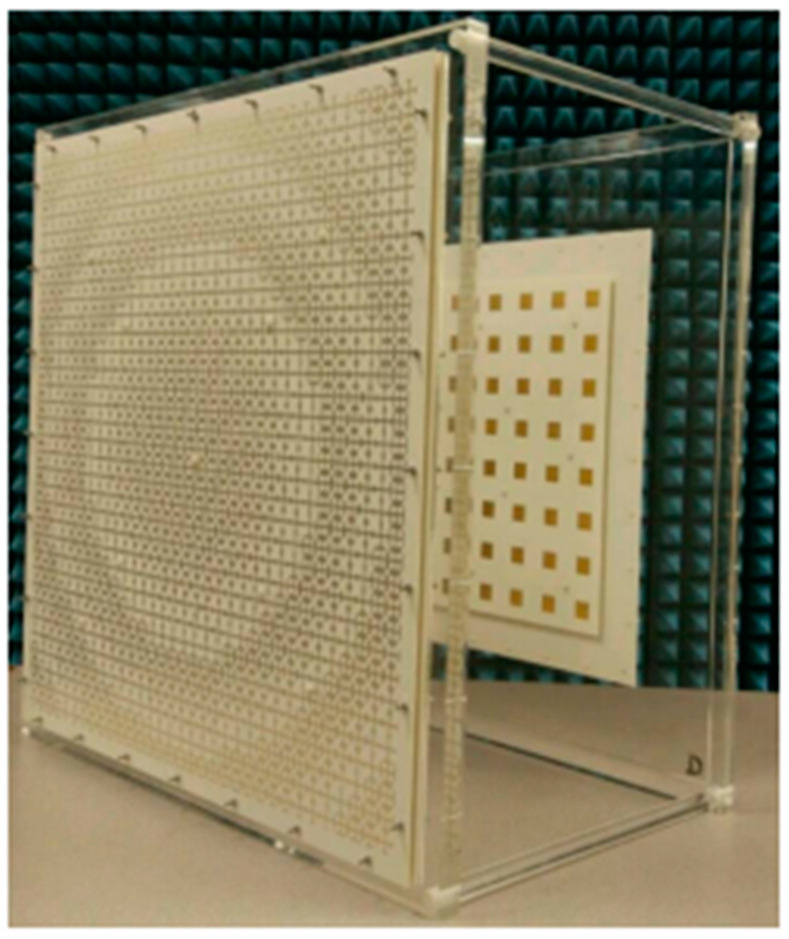
Full-dimensional massive MIMO of a planar (8 × 8) dual polarization antenna array feeding a meta surface lens antenna [46].

On the other hand, a suggested and characterized meta surface lens antenna was fed through a dual polarization plane (8 × 8) antenna array for sub-6 GHz full-size array MIMO and multi-beam systems, as demonstrated in Figure 9. A lightweight multilayer meta-surface structure built of Jerusalem transverse elements was used to build the planar lens. An (8 × 8) dual-polarized stacked patch array fed a lens antenna with dimensions of (560 mm × 560 mm × 266 mm) to operate at the range of 5.17–6.10 GHz. With a maximal gain of 22.4 dBi and a 3.3 dB at 5.6 GHz gain variation, the scanning range of ±25° was attained. The beam coverage characteristics, pattern envelope correlation coefficient (ECC), and port-to-port isolation properties indicate that it can provide full-dimensional access. Additionally, while isolated beams can achieve frequency division multiplexing, beams operating at the same frequency offer a high-gain multi-beam antenna system [46].

A highly integrated active multi-beam antenna system was developed and implemented at 5.8 GHz with 64 RF channels and 256 antenna elements for high-volume 5G MIMO wireless communication applications. The 64-channel highly integrated active multi-beam antenna system provides a testing ground for the digital beamforming algorithms and the mMIMO channel estimation for 5G wireless communication. Eight PCB boards with six layers make up the 64-channel multi-beam antenna system. The 256-element antenna arrays of the 64-channel multibeam antenna system could be assessed with an array gain of 18 dB. Each PCB measures 320 mm in length and 215 mm in width. The beam sweep of 64 × 4 antenna arrays is between −30° and 30° with 10° intervals [47].

The authors in [48] provided a 3D coverage optimization approach after analyzing the structure of a 128 elements/64-channel active mMIMO antenna array. There were 64 dual-polarized elements (8 × 8) in the antenna array, with every one of the elements ending in a +45° and a −45° polarized antenna. The total 3D beamforming and coverage optimization regarding the array were realized by integrating and optimizing two major plane patterns of the desired 3D radiation pattern. Additionally, the simulation verified and validated the 3D beamforming optimization approach, demonstrating its effectiveness.

#### 2.1.3. Combined (Sub-6 GHz/MM Wave) mMIMO

There are three scenarios for 5G, where enhanced mobile broadband (eMBB) is one of them. eMBB employs mMIMO with multiple antennas. 5G uses frequency candidates below 6 GHz and below 28 GHz. The principal objective is to model large-scale MIMO antenna systems operating at 3.5 GHz and 26 GHz. The antenna in [49] included a rectangular patch shaped like an array, with 12 patches for 3.5 GHz and 96 patches for 26 GHz, for 108 patches on the antenna. The constructed antenna will be utilized as an internal transmit antenna. This antenna has a 2.2 dielectric constant proximity-coupled feed and a connection. The designed antenna has an s-parameter result of <−10.8199 dB, a mutual coupling of <−32.6201 dB, and a 7.3 dB gain [49].

In the mMIMO system, increasing the number of elements increases the channel capacity. Various research has explored the effect of the number of antennas while assuming uniform antenna gain across very large arrays, relying mostly on channel properties to investigate the influence of edge effects and mutual coupling on the variation in the gain illustration in a 32-element large-scale MIMO array. The impact analysis focused on contrasting patch and dipole antennas, which are typical of the antennas frequently used in the high-volume MIMO experiments performed today. Compared to a dipole array, the finite patch array has a lower gain pattern variation. The massive MIMO system is affected by a large gain pattern variation in that not all antennas contribute equally to all users, and the number of effective antennas seen for a single user is decreased. As a result, as seen at the system level, the zero-force MIMO detector for all users is reduced in speed by 20% for patch arrays and 35% for dipole arrays. On the other hand, combining maximum ratios creates inequality and injustice in users. Antenna measurements depend on measuring 32 active elements in the array, which can be achieved using a large MIMO test bench installed in an anechoic chamber. In terms of system throughput, patch arrays have proven to be the best option. The 31 mm square patch on the microstrip model had two combined U-slots that were each 1.4 mm wide. Next, the 2.4–2.62 GHz and 3.4–3.6 GHz frequency bands were covered. At 2.6 GHz, the main comparison was performed. It has been demonstrated experimentally that the gain patterns of the various antenna elements in a finite array exhibited significant variation. The edge effect and mutual coupling are both responsible for this beamforming, which is highly dependent on the angle of arrival. The gain variation is greater in a dipole array because the system has more powerful mutual coupling. As a result, the array composed of omni-directional elements is more angle-sensitive than a patch array comprised of directional elements [50].

The two-user MU-MIMO (multi-user MIMO) capacity based on a zero-forcing block diagonalized (ZFBD) scheme, and the distribution of eigenvalues were examined in a viable urban macro (UMa) environment at 3.5 GHz. It covered the topic of expanding the amount of Tx items from 8 to 256. For Tx numbers up to 64 and 128, respectively, ratios of over 63% and 73% were attained in comparison to independent channels with the same distribution (i. i. d), with only a slight improvement seen when the numbers rose quickly. Additionally, when the number of Tx rose, there was a very slight tendency toward a uniform distribution of eigenvalues. Compared to traditional MIMO, mMIMO produces more ordered sub-channels and de-correlated user channels, but (i. i. d.) channel gaps still exist in the measuring environment under consideration. As shown, capacity grows with the number of antennas installed per user, and the performance is influenced by the antenna layout on both the receive and transmit sides. As a result, in a measurement scenario, orthogonal user channel representation is not used [51,52].

Massive MIMO is a leading technology contender for the 5G mobile communication system because it can fully utilize the existing space resources, significantly increase the spectrum efficiency, and lessen the current spectrum resource crisis. It is required to record various radio channel characteristics from the current MIMO system to study mMIMO technology. Thus, a 3.33 GHz measurement campaign was carried out outdoors, utilizing a sizable virtual antenna array that included 64 components. According to the measured data, common channel parameters including the azimuth power delay spectrum (PADS), power delay profile (PDP), azimuth power spectrum (PAS), power delay spectrum (PDS), azimuth spread (AS), and delay spread (OS) behaved as expected. In both the latency and spatial domains, the acquired data demonstrate that channels are not static for large array sizes. The outcomes can be used to create mMIMO channel models and construct 5G communication systems [53,54].

For upcoming 5G applications, a 16-port, low-cost MIMO non-planar antenna system was constructed on a 3D octagonal polystyrene block. The bottom and top of the polystyrene block were visible due to the arrangement of the MIMO elements on the eight sides of the octagonal block. One of the antenna’s components was a slotted microstrip patch with a broken ground plane and a step-biased feed line. The frequency range (3.35–3.65 GHz) for every one of the MIMO elements was constructed on an FR4 substrate with dimensions of 22 mm × 20 mm for 5G applications. A meander line-based, exponential, near-zero, negative (NZI-ENG) metamaterial decoupling structure was used to increase the isolation between array members. Ground planes and isolation structures for common connections were positioned in the bottom layer, whereas array elements were positioned in the top layer [55].

The antenna elements were isolated by >28 dB in side-by-side configurations thanks to the metamaterial-based decoupling structure. The envelope correlation coefficient (ECC) < 0.10, total active reflection coefficient (TARC) < −18 dB, and channel capacity loss (CCL) < 0.30 were all within adequate bounds. The suggested non-planar 3D-MIMO antenna system could be utilized for wireless personal area network applications and indoor positioning systems in the case when different 5G devices are connected by wireless means to a central server [56]. The spatial multiplexing feature of a typical 5G MIMO system was realized by combining several antennas at the receiver and transmitter. The 3.3–6.0 GHz band-capable proposed broadband 16-element indoor BS antenna array was made for 5G applications. In order to cover the lower and upper bands (LTE bands 42/43/46-N77-N78-N79), a monopole antenna in the shape of a was used. A printed hexa-kaidecagon polygon, which was close by on a substrate, provided the antenna element. When the antenna elements were in a position that provided good polarization diversity and good isolation, the planned BS array was designed, constructed, and tested by examining the overall outcomes, antenna performance, S-parameters, and radiation patterns. For MIMO performance testing, it also achieved a high antenna efficiency of roughly 82–93.2% and a very low ECC (envelope correlation coefficient) of <0.02. The (16 × 16) MIMO system’s expected ergodic channel capacity was 85 bps/Hz [57]. In this study, all designs were used in the fabrications, and the values were in measurements. Table 1 shows the enhancement and support of mMIMO techniques for base station applications. Table 2 compares the 5G sub-6 GHz mMIMO antenna techniques.

Many parameters characterize the pattern of large-scale MIMO antenna systems such as MEG, DG, TARC, and ergodic channel capacity. This study focused on the critical performance-enhancing parameters that reflect the performance of the antenna system.

### 2.2. 5G Smartphone mMIMO Array Antenna Techniques

Many techniques support massive MIMO systems in smartphone applications working in the sub-6 GHz bands, which are classified depending on the number of elements.

#### 2.2.1. 18-Port 5G Massive MIMO

**Figure 10:**
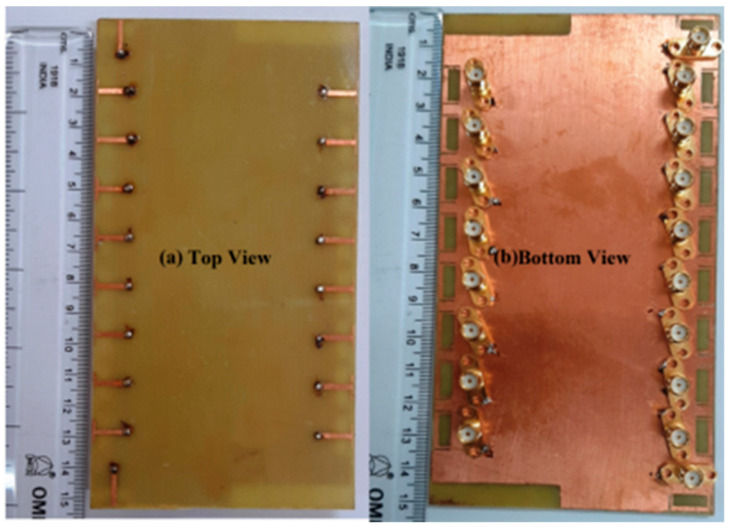
18-element antenna system of the slot antenna with decoupling open-ended slots [58].

The mMIMO/diversity (4G/5G) model of the 18-element antenna system was created at sub-6 GHz long-term evolution (LTE-42/43) bands of 3.4–3.6 GHz and 3.6–3.8 GHz, respectively. By using a straightforward slotted antenna with an open slot as the radiator, a small design could be achieved. Additionally, this slot serves as a decoupling component to enhance isolation between other elements. The antenna array elements were constructed on a low-cost FR4 substrate with dimensions of 150 mm × 80 mm × 1.6 mm, frequently utilized for 6-inch smartphones, as shown in Figure 10. Antenna gain of >5.3 dBi, impedance matching (coefficient of reflection >20 dB), total efficiency (>87%), port isolation (>20 dB), and envelope correlation coefficient (<0.010) over operating frequency were demonstrated by the simulation and measurement results. The performance metric of the MIMO antenna was verified by computing the capacity of the ergodic channel using the Kronecker channel model [58].

#### 2.2.2. 12-port 5G Massive MIMO

**Figure 11:**
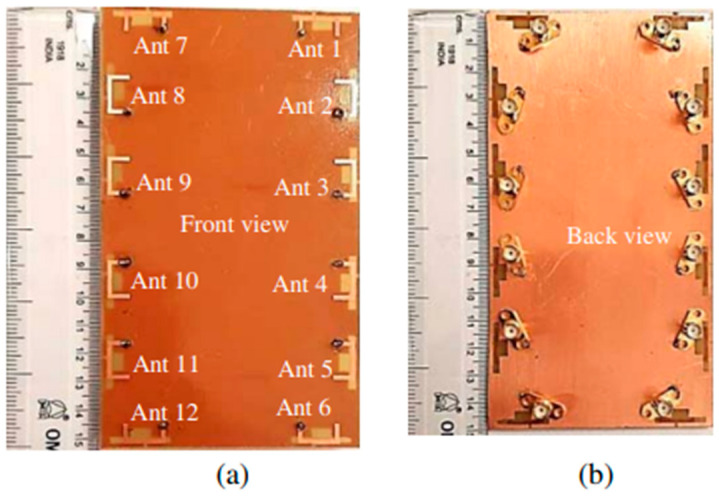
Fabricated prototype of 12-port, (**a**) Front view. (**b**) Back view [59].

A 12-port LTE antenna array (42/43/46) for 5G high-capacity networks up to 6 GHz, 3400–3600 MHz, 3600 MHz–3800 MHz, and 5150 MHz–5925 MHz was used. The three different antenna element types that make up a MIMO smartphone application are: an inverted -shaped antenna, a shorter inverted L-shaped open slot antenna, and a longer inverted open slot L-shaped antenna. Figure 11 illustrates eight antenna elements for MIMO in LTE 42/43 band and six antenna elements for MIMO in LTE 46 band (12). According to the actual measurement results, the LTE frequency band (42/43/46) had a reflection coefficient that was better than −6 dB, an isolation of <−12 dB, and an overall efficiency of >40%. Additionally, the suggested antenna array displayed good MIMO performance in LTE bands 42/43 and 46, with envelope correlation coefficients of 0.15 and ergodic channel capacity of over 34 b/s/Hz and 26.5 b/s/Hz, respectively [59].

#### 2.2.3. 10-Element 5G Massive MIMO

**Figure 12:**
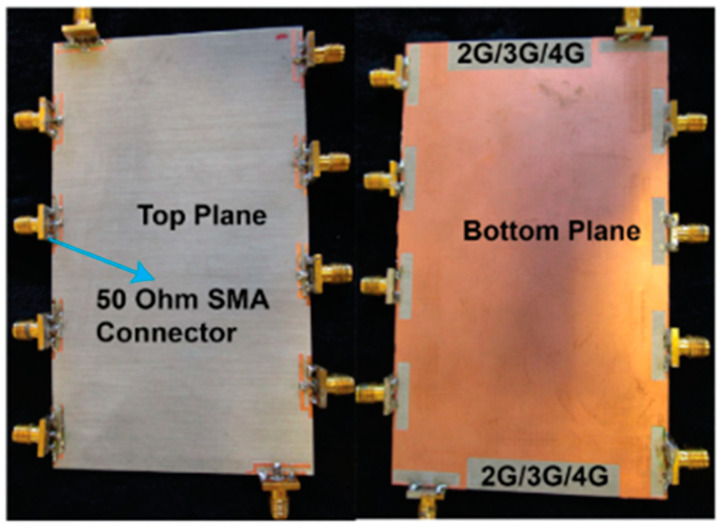
Prototype of the (10 × 10) MIMO antennas with the IFS fed by end launch 50 Ω connectors at the top layer and slots at the bottom layer [60].

Wide impedance bandwidth (IBW) (5G), multi-antenna, 10-element, sub-6 GHz, and mMIMO terminals are advised. The hybrid loop antenna element’s inverted-F stub is fed by a grounded coplanar waveguide. To generate wider IBWs, multiple resonant modes are merged by tuning. As illustrated in Figure 12, we selected an antenna element location around the edge of a standard smartphone backplane (120 mm × 70 mm). Two elements were positioned along the horizontal edge and orthogonal to the other elements; however, they were positioned along the vertical edge to enhance the variety performance. An experimental antenna with a large impedance bandwidth (−6 dB) centered at 63% at 4.68 GHz was constructed and measured. The isolation between measurements was at least 18 dB. The simulated envelope’s most significant correlation coefficient was 0.21, and its minimum antenna effectiveness was 78.4%. Each component was printed on a Rogers 4003 substrate that was 1.52 mm thick and measured 17.2 mm × 3.8 mm in size. The suggested multi-antenna terminal is suited for LTE bands (42, 43, and 46) and 5G sub-6 GHz (3.2–6.1 GHz) communications [60].

**Figure 13:**
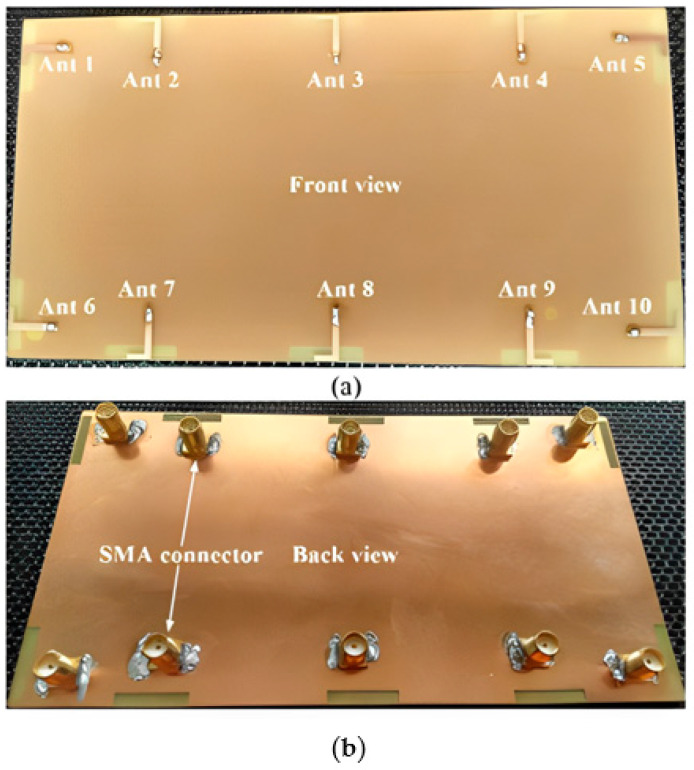
Fabricated prototype of the (10 × 10) MIMO of T-shaped, coupled fed-slot antenna elements. (**a**) Front view, (**b**) back view [61].

A future 5G smartphone will feature a multi-band, 10-antenna array to support mMIMO applications in the sub-6 GHz spectrum (LTE bands 42–43–46), as shown in Figure 13. To implement the (10 × 10) MIMO in all three bands of the LTE applications, 10 T-shaped, coupled fed-slot antenna elements with the capacity to excite dual resonant modes were incorporated on the system board. These parts were equipped with spatial and polarization diversity approaches to enhance isolation and reduce the coupling effects. The performance of the suggested antenna array prototype was assessed in the lab. In the high and low bands, the antenna efficiency was evaluated as desirable above 62% and 42%, respectively. For a LTE band (42/43/46) and a 10 × 10 MIMO system, the maximum ergodic channel capacities were found to be 48 bps/Hz and 51.4 bps/Hz, respectively [61].

#### 2.2.4. 8-Element 5G Massive MIMO

For 5G mMIMO systems, a multi-band antenna array has been studied in the literature as a potential solution. The antenna discussed here is suited for use in multi-mode operations since it creates the feature of polarization diversity and operates on multiple bands. Eight modified planar-inverted F antenna (PIFA) elements make up its configuration, and each one is placed in a distinct location on the smartphone’s main board. To simplify the integration process and design, both the ground plane and antenna elements were etched on the same layer. For the S11 ≤−10 dB performance, the PIFA elements of the MIMO design operated at frequencies of 2.50–2.7 GHz, 3.40–3.8 GHz, and 5.60–6 GHz, spanning the LTE (2600, 42/43/47) operation bands, respectively. The proposed design can support vertical or horizontal polarization thanks to the positioning of the antenna elements. The investigation focused on the fundamental characteristics of the design that was proposed, which possessed sufficient efficiency, good S-parameters, acceptable isolation, and provided coverage for dual-polarized radiation. In addition, the results of the modified PIFAs’ TARC and ECC calculations showed that they had a low value across all of the operation bands [62].

**Figure 14:**
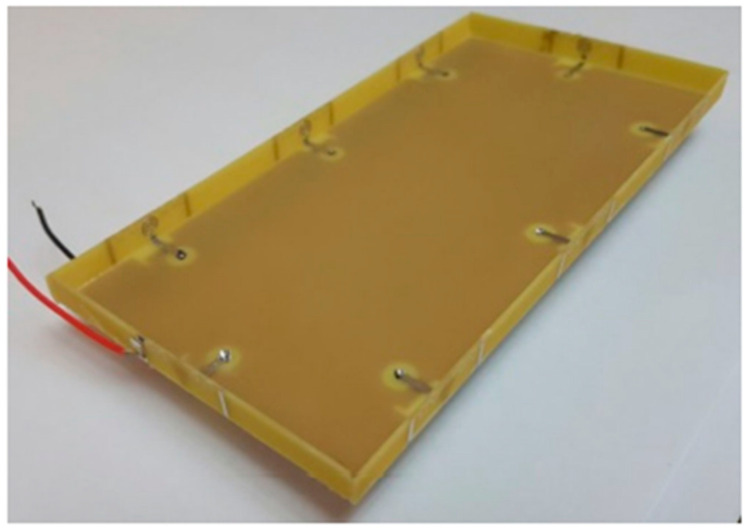
Reconfigurable (8 × 8) MIMO frame–antenna structure [63].

In order to provide coverage for two bands of frequency—an LB for LTE2500 and an HB for the next 3.5 GHz standard with the diversity of the polarization—a multi-band re-configurable structure of the (8 × 8) MIMO frame antenna is presented. LTE2500 might make use of the low band. The coming standard would take advantage of the high band. For forthcoming (4G/5G) mMIMO smartphone applications, a switchable antenna array is described. Two antenna array modules were used to create the prototype: a switchable (6 × 6) MIMO 5G antenna module with pattern diversity and a (2 × 2) MIMO 4G antenna module with frequency and circular polarization diversities. These two modules together make up the antenna array. A micro-strip line with a tuning slot on the ground plane supplies each element in the 5G and 4G modules. This line is a parasitic element on a non-metal frame that includes a spiral patch as well as a short-ended, C-shape stab. This slot is utilized to change the line’s frequency. Each element occupies a space on the ground plane that is 22 mm × 9.3 mm in size, as illustrated in Figure 14. These antenna elements employ spatial variety and polarization approaches to decrease the coupling effects and improve isolation. The intention behind doing this is to obtain the desired outcomes. By incorporating the PIN diode into the antenna element’s structure, the module regarding a 4G antenna may operate and switch between the two 5G bands and LTE-2500 operating bands. The module of a 5G antenna can operate in a frequency range of 3.4 GHz–3.6 GHz and might be able to meet the needs of a future 5G application. The suggested antenna has an isolation level higher than 15.2 dB in both the OFF and ON states of the PIN diodes, which can be accomplished without the use of decoupling structures [63].

#### 2.2.5. 4-Element/8-Port 5G Massive MIMO

Massive MIMO systems for 5G have one proposed design for an antenna array for mobile phones that uses diamond-ring slot elements. Four double-fed (eight ports/four elements) diamond-ring slot antenna elements make up the design configuration, and they are each placed in a separate corner of the printed circuit board for the mobile phone (PCB). A cheap FR4 dielectric with overall dimensions of 75 mm × 150 mm was utilized as the design substrate. The elements of the antenna were fed by L-shaped micro-strip wires with a resistance of 50 ohms. Because of the orthogonal positioning of the micro-strip feedlines, the diamond-ring slot elements were able to display the radiation and polarization pattern diversity characteristics. For each antenna radiator, we were successful in achieving a good impedance bandwidth with a range of 3.2–4 GHz (S11 ≤ −10 dB).

Nevertheless, this value was 3–4.2 GHz when S11 is ≤−6 dB. The design that was proposed offers the necessary radiation coverage for 5G mobile phones. The proposed MIMO smartphone antenna has achieved high levels of isolation and efficiency. Additionally, for the entire band of interest, the TARC and ECC of the antenna elements are incredibly low, which guarantees that multi-antenna systems under consideration could be applied to large MIMO and diversity applications [64].

Even though the number of elements of massive MIMO in smartphones should be equal to or greater than eight elements, many technologies do not support the mMIMO system in smartphones, despite having the same number of mMIMO elements. However, these technologies can be considered as good candidates for the massive MIMO system.

The performance varies depending on how many antenna elements (AEs) are combined into a single MIMO mobile terminal. This is carried out in free space when a user is holding the mobile terminal in their hand while in data mode. The performance of the MIMO terminal was evaluated with regard to efficiency, ECC, multiplexing efficiency, capacity, and maximal ratio combined with two additional AEs each (as many as 18 AEs). The investigation began with a minimum of two AEs and progressed to 18 AEs (MRC). For use with 5G technology, the integrated MIMO antennas were identical and had a frequency range of between 5 and 6 GHz. According to the findings, the ECC rose along with the total number of adverse events. In spite of this, the ECC for the case of 18 AEs was still lower than 0.32 in both free space and when a user’s hand was present. In the meantime, it was observed that the free space efficiency, which was approximately 90% for the two AEs, decreased with the increasing number of AEs, reaching approximately 50% with 18 AEs. However, the effectiveness of the elements shifts depending on the user’s hand because each element interacts with the hand. The efficiency of some AEs dropped as low as 5% when the hand was used to block them directly, while the efficiency of other AEs remained at up to 75% throughout the experiment. When a center frequency was 5.5 GHz, the free space capacity became 11.10, 49.50, and 83.2 bit/s/Hz with 2, 10, and 18 AEs, respectively. These levels decreased by 11% (two adverse events), 35% (10 adverse events), and 31%, respectively (18 AEs), when a mobile terminal was held close to the user’s hand. The influence of poor AE correlation was shown to be a negligible component, and multiplexing efficiency demonstrated that a decline in the efficiency of the AEs mostly drove the deterioration of the capacity. This was discovered to be the case after it was established that the effect of the AEs’ poor correlation was a negligible factor. Additionally, the maximal ratio combining the method’s gain, capacity, and diversity gain was examined [65].

A mMIMO array with 10-ports or 14-ports is one of the designs that has been proposed for use in 5G mobile phone applications. The LTE band 42 (3.4–3.6 GHz) antenna, a LTE band 43 (3.6–3.8 GHz) antenna, and a LTE band 46 antennae are to be covered by a dual-band ring loop antenna (5.15–5.925 GHz). The proposed arrays are designed to change the battery position and exploit the space to add more antennas. To achieve higher isolation, the substrate is formed as the loop antenna elements are printed on a separate dodecagon FR4 substrate with different orientation angles. The proposed designs can achieve an isolation better than −26 dB. The envelope correlation coefficient (ECC) based on S-parameters was found to be better than 0.005 in LTE band 42/43 and 0.006 in LTE band 46. Additionally, the ECC was evaluated based on far-field radiation patterns and was found to be less than 0.2 in LTE band 42/43 and less than 0.12 in LTE band 46. The channel capacities were attained, the 10 × 10 MIMO achieved 57.6 bps/Hz, and the (14 × 14) MIMO achieved 72 bps/Hz. Additionally, the specific absorption rate (SAR), diversity gain (DG), and the effect of frame insertion on the proposed array were also discussed [66,67].

The next-generation 5G smartphone offers a small broadband and dual-band antenna array that forms a 12-element MIMO array. Each antenna uses a coupling feed to improve isolation from other antenna elements nearby and impedance matching. With a hybrid loop antenna/IFA, six 5G MIMO antennas were designed to cover the 3.3~4.2 GHz frequency range. To span 2.4~2.5 and 5.1~5.9 GHz, six WiFi MIMO antennas work as a coupled-fed dual-loop antenna. With very little ground clearance, the simulated results showed excellent isolation, impedance matching, and antenna efficiency capabilities [68]. Future MIMO operations in smartphones for 5G will use a tri-polarized 12-antenna array that operates in the 3.5 GHz band (3.40–3.60 GHz). The mutual couplings will be reduced, and the design procedure is made simpler by using the orthogonal polarization technique. A QMSIW antenna and two open-end slots could be combined to create a small (17 mm × 17 mm × 6 mm) three-antenna tri-polarization block that operates in a 3.5 GHz band. The antenna has an incorporated quarter ode substrate waveguide as a result of this configuration. The three antennas in the block might have low mutual coupling and strong impedance matching due to the presence of orthogonal polarization properties. Next, combining such types of tri-polarization blocks into four different arrays, a 12-antenna MIMO array was designed for smartphone applications. With just two additional decoupling structures, the presented array might achieve low correlations and good isolation between antennas.

Additionally, a tri-polarization feature incorporated into the design is to blame for this. Good antenna performance is conceivable including a return loss of more than 10 dB, isolation of more than 12.5 dB, and antenna efficiencies of over 50%. The channel capacity of the 12-antenna array has been determined to be around 57 bps/Hz in a (12 × 12) MIMO system with a 20 dB SNR, showing that the suggested array using the tri-polarization approach is a solid candidate for potential future 5G terminals [69].

**Figure 15:**
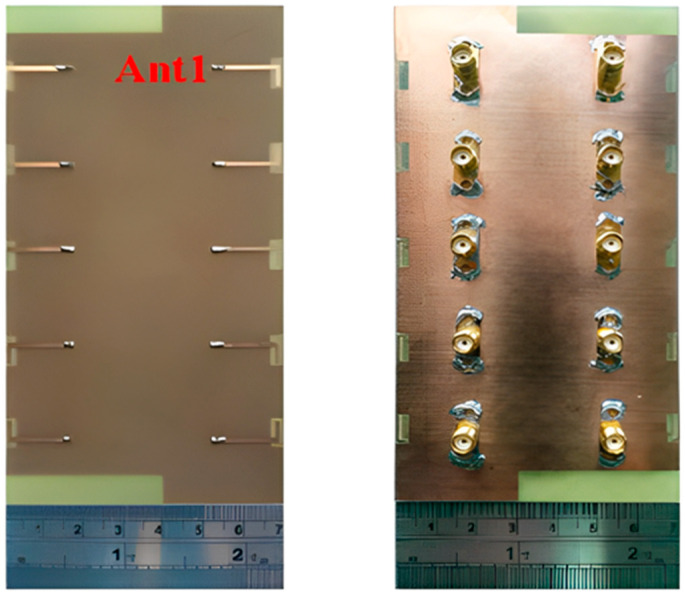
Fabricated (10 × 10) MIMO array with the microstrip line-fed open-slot antenna [70].

In order to facilitate multi-input multi-output (MIMO) functionality in a mobile device, a 10-antenna array that operates in the 3.6 GHz band (3.4–3.8 GHz) has been structured. The proposed antenna array will consist of microstrip line-fed open-slot antennas, each of which will have the same modest dimensions of 3 mm × 8 mm. The proposed array is made up of two sets of five antennas, each of which is arranged symmetrically along one of the smartphone’s long side edges of the system ground plane. The suggested array is predicted to be positioned in the constrained space between the display screen and longitudinal side borders of a smartphone, as seen in Figure 15. Any two antennas in the suggested array were found to be capable of achieving a satisfactory ECC of 0.1 and an adequate level of isolation (more than 10 dB). The proposed array’s maximum channel capacity in a (10 × 10) MIMO system has been estimated to be around 47 bps/Hz at a SNR of 20 dB. This exceeds the upper limit of the ideal (2 × 2) MIMO system, which is 11.5 bps/Hz, has 100% antenna efficiency, and has no ECC between the antennas [70].

Two radiators were etched on the outer and inner surfaces of the side-edge frame device to form a dual-band 10-element MIMO array based on dual-mode inverted-F antennas (IFAs). This array was designed for use in 5G terminal applications. An additional one-quarter wavelength mode was radiated by the inner part of the antenna at 4.9 GHz, while the outer part of the antenna was responsible for generating the low-order mode at 3.5 GHz. The IFA can accomplish dual-band operation in this manner despite its compact size of 10.6 mm × 5.3 mm × 0.8 mm. In order to support 5G terminal applications, a dual-band, ten-element multiple-input multiple-output (MIMO) array was developed. The foundation of this array is the suggested antenna. Combining decoupling branch structures with neutralization line structures helps improve isolations between the elements. The idea for a prototype of a 10-element MIMO array was developed, built, and tested to confirm the practicality of the design concept. The 3.30–3.6 GHz and 4.80–5 GHz frequency bands can both be covered by the suggested antenna with high efficiency and strong isolation, according to the experimental results. To evaluate the effectiveness of MIMO for 5G applications that operate below 6 GHz, the ECC and the channel capacity were also evaluated [71].

**Figure 16:**
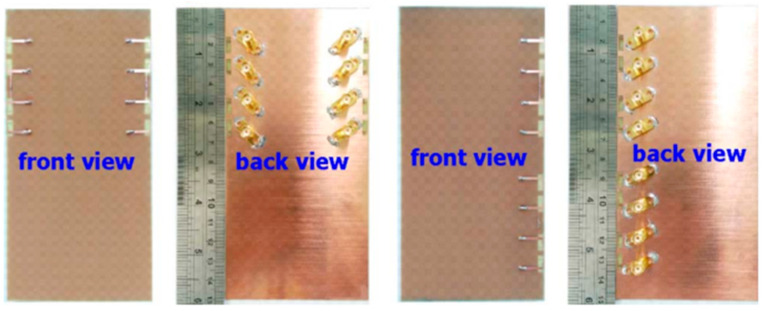
The eight- and sixteen-antenna MIMO arrays formed using the quad-antenna linear (QAL) array as a building block with front and back views [72].

The quad-antenna linear (QAL) array can be used as a building block for smartphone 16-antenna and 8-antenna arrays for 3.5 GHz MIMO operation. The QAL array has the dimensions of 50 mm × 3 mm. Two QAL arrays are positioned on the system circuit board of the smartphone, either on two different sides or on the same side, to produce an 8-antenna array. The analysis of the 16-antenna array made up of four QAL arrays arranged along two opposing side edges is shown in Figure 16. With a 20-dB SNR, the calculated channel capacity for use in a (16 × 16) MIMO system can reach approximately 66–70 bps/Hz. The achieved channel capacity is about 5.70–6.10 times more than the maximum achievable in an ideal (2 × 2) MIMO system with antennas operating at 11.5 bps/Hz (100% antenna efficiency) [72].

**Figure 17:**
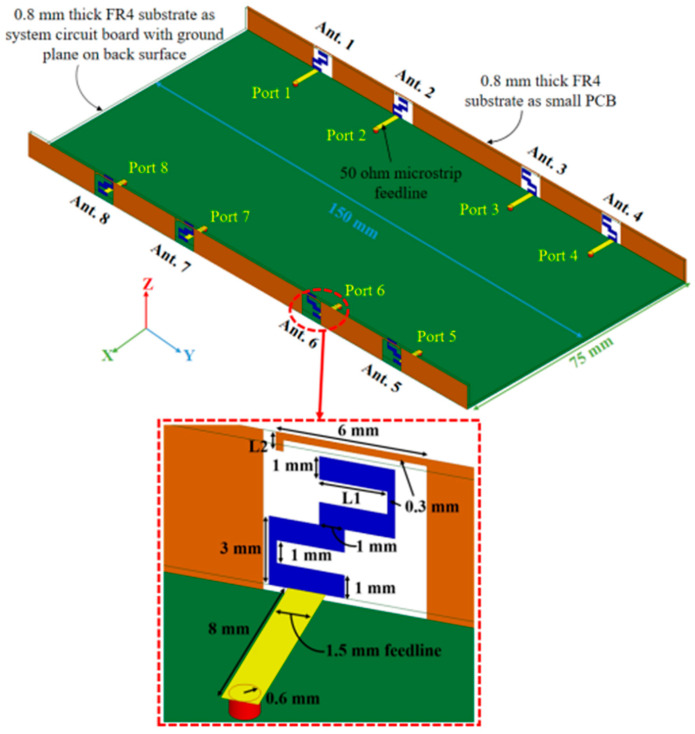
Dimensions and specifications in millimeter of the proposed triple-band, eight-element antenna array (**top**) Prospective view; (**bottom**) Side view [73].

For applications on triple-band 5G metal-frame smartphones, the author in [73] proposed a MIMO antenna array with good performance whereas the structure use an L-shaped radiation strip and an S-shaped feeding strip (Figure 17). The achieved bandwidth increased for lower frequency band while minimizing size by employing an S-shaped feeding strip. The novel aspect of this investigation is the antenna element’s small size with 6.5 mm 7 mm construction. The suggested eight-antenna array’s 6 dB impedance bandwidth may completely cover the 3.3–3.8 GHz, 4.8–5 GHz, and 5.15–5.925 GHz frequency bands.

A 0.8 mm thick, low-cost, FR-4-substrate with dimensions of 136 mm × 68 mm and an inverted L-shaped, eight-element MIMO antenna system was developed. This resonates at 3.50 GHz with a measured bandwidth of 450 MHz, inter-element isolation of over 15 dB, and gain of 4 dBi. The system was developed on an inexpensive FR4 substrate. A total of eight elements in the shape of an inverted L and parasitic L-shaped strips extending from the ground plane make up the proposed design. These short stripes served as tuning stubs for four inverted L-shaped monopole elements on the side of the chassis. To achieve the objective of reaching the necessary frequency range, the electrical length of antennas was increased. The decision was made to create a prototype, and the findings of the investigations showed that there was adequate impedance matching and reasonable measured isolation within the intended frequency range. MIMO performances such as the ECC and MEG were also determined. Because of how straightforward its form is and how thin it is, it has the potential to serve as the chassis for future handsets. As a result, different user hand scenarios such as single-handed and dual-handed use have been investigated. Along with the SAR, a discussion was held regarding the effects of various hand scenarios on various MIMO parameters. The fact that the proposed system worked well in different situations shows that the proposed structure has a good chance of being used in the next generation of radio smartphones [74].

For 5G applications operating at sub-6 GHz, a new smartphone array antenna design that utilizes new double-fed CPW-fed resonators was comprised of two modified, T-ring radiators that were closely spaced and operated in a frequency band that ranged from 3.3 to 4.4 GHz. An (8 × 8) MIMO antenna was formed by placing four pairs of CPW-fed diversity antennas in each of the board’s four corners that make up the smartphone. Without needing an additional decoupling structure, it offers excellent isolation of greater than −16 dB. The design of the CPW-fed smart phone antenna array occupies only a very small portion of the board, which is partly due to the fact that the array itself is quite small, and partly because the elements that comprise the array are placed in very particular locations. The proposed MIMO design not only has enough radiation coverage to support all of the main board’s sides, but it also has different polarizations [75].

Future smartphones will have a MIMO array with eight unique, balanced, open-slot antennas that operate in the 3.50 GHz band (3.40–3.60 GHz). With less ground design, this antenna’s balanced slot mode could improve the isolation between two neighboring input ports. The 3.5 GHz band is where the array operates. By carefully planning the positions related to the eight antenna elements that further reduce the coupling between the elements of the antenna, it is also possible to efficiently reach the desired polarization variety. The positions of antenna elements could be carefully arranged to achieve this. There was good impedance matching (return loss > 10 dB), high total efficiency (>62%), high isolation (>17.5 dB), and a low envelope correlation coefficient (ECC0.05) across the necessary operation bandwidth, according to the tests. To verify the effectiveness of MIMO, calculations regarding the ergodic channel capacity were made with the use of the Kronecker channel model. Investigations into the hand phantom’s effects were also conducted [76].

For 5G MIMO metal-rimmed smart phones, the combination of the orthogonal monopole/dipole modes in a lower band and orthogonal slot/open-slot modes in a higher band was reported. This led to a wideband, orthogonal mode, decoupling property of a dual antenna pair with a shared radiator. For 5G MIMO smartphones, this property was realized. The dual-antenna pair can show a large impedance band width of 3.30–5 GHz, in addition to a strong isolation of >21.0 dB across the whole band thanks to the orthogonal-mode design method without the need for any additional external decoupling devices. Without the need for any directional couplers, this is accomplished. The simulation and measurement results showed that an 8 × 8 MIMO system can fulfill the proposed edges of the smartphone if four of these dual-antenna pairs are arranged on each of the two sides of the smartphone. Both 8 × 8 MIMO systems have the potential to provide greater than 12.0 decibels of isolation between all ports and an envelope correlation coefficient of <0.11. The two antenna elements that make up the dual-antenna pair have average efficiencies of 74.7% and 57.8%, respectively, according to the measurements taken. The proposed design has the benefits of a shared radiator, a wide bandwidth, and the ability to work with metal rims and so could be used in 5G phones in the future [77,78].

A dual-band (8 × 8) MIMO array antenna that operates in the 3.5 GHz band (3.4–3.6 GHz) and the 5.5 GHz band (5.150–5.925 GHz) for 5G mobile handsets was made up of a comb-shaped monopole and an L-shaped open slot antenna that were symmetrically positioned on the inner surface of the side-edge frame of the smartphone. It is feasible to achieve pattern diversity, which could lower the ECCs and enhance the functionality of MIMO systems. The results show that the isolation of 15 dB and 10 dB in the high and low bands, respectively, could be accomplished without introducing any additional decoupling element and that the desired bands could be satisfied with an impedance matching of 6 dB. According to the findings, the suggested MIMO antenna will be a fantastic option for 5G services in the near future [79].

MIMO schemes with 4-antennas/8-dual polarized ports were developed on a printed circuit board (PCB) side (67 mm × 139 mm), side by side, using an epsilon 4.4, FR-4 dielectric substrate, and the thermal conductivity was 0.025. To improve the radiation characteristics, circular slot radiators were etched into the substrate. In addition, to reduce mutual coupling, two rectangular open-fin parasitic radiators were included into a single square-slotted radiator in the suggested MIMO architecture. The proposed single-antenna model had an impedance bandwidth of −10 dB and covered the frequency band 5.81–6.66 GHz. However, at −6 dB, the bandwidth rose to 1.47 MHz (5.48–6.95 GHz). According to the data, a return loss of −20 dB is feasible, and the isolation of the dual micro-strip lines could reach −45 dB. The suggested antenna could be included in new smart mobile devices for upcoming 5G wireless communications [80].

Table 3 presents the massive MIMO technique enhancement and supposition for smartphone applications. Table 4 compares the previous references for massive MIMO for smartphone applications.

Higher antenna efficiency is needed for mobile devices using mMIMO systems. This entails adding additional antennas to the same physical area or increasing the bandwidth of already installed antennas. The suggested system’s effectiveness was examined by adjusting critical operating parameters including ECC, MEG, scattering parameters, and channel capacity, and carrying out research such as user hand analysis. The technology is safe to use close to the human body, according to a SAR analysis that was conducted to understand how it interacts with the body.

## 3. 5G Antennas as a Candidate for Massive MIMO Technique at Sub-6 GHz

**Figure 18:**
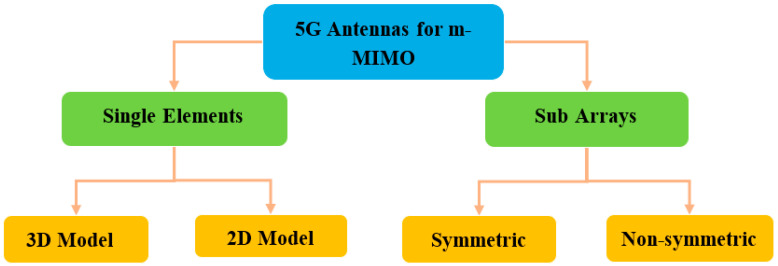
5G antenna design techniques for 5G massive MIMO at sub-6 GHz.

An essential component of wireless communication systems is the antenna. Wireless systems that use a large number of antenna elements/arrays at both the transmitter and receiver are called massive MIMO. 5G antennas enable multiple-input multiple-output (MIMO). Various 5G antennas at sub-6 GHz designs are considered as candidates for developing massive MIMO antenna arrays. Figure 18 shows several structures of 5G antennas can be summarized in the block diagram below.

### 3.1. Single Elements

Single-element antennas are easy to design, implement, and manufacture. There are two types of 5G designs to reduce the size of antennas: three-dimensional designs and two-dimensional crossed dipole and patch antenna designs, as discussed below.

#### 3.1.1. 3D-Model Antennas

An experimental study was created on a dual-polarized broadband BS antenna with a new feed structure. The suggested antenna includes two crossed pairs of dipoles, two specifically designed feed connectors, a carrier (also known as a balun), two dielectric pads, and a reflector. We created a prototype and evaluated it to ensure the designed antenna worked as intended. The antenna achieved a port-to-port isolation of over 32.5 dB from 3.14 GHz to 5.04 GHz, a bandwidth of roughly 46.5%, and a reflection coefficient of <−15 dB. With half power beam width values of 71.8° ± 2.5° in the horizontal as well as the vertical planes and a gain of roughly 8 dBi in the operating band, it also offers a remarkably stable radiation pattern. The suggested antenna is also appropriate for BS in the sub-6 GHz frequency band of 5G cells, thanks to its properties [81].

**Figure 19:**
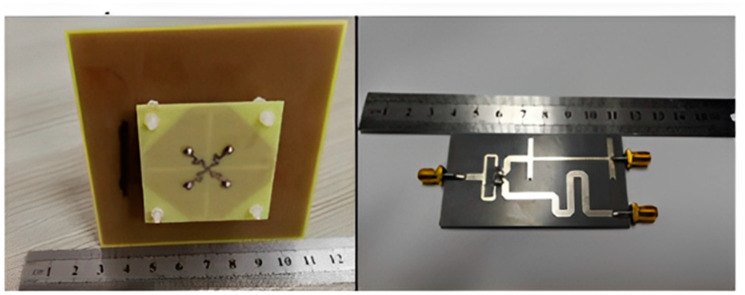
Fabrication of dual-polarized slot antennas with octagonal patches and folded feedlines [82].

For BS applications, a low-profile, differentially fed, dual-polarization slot antenna was suggested. Its radiator is an octagonal patch that has 2-conventional H-shaped slots etched into it. A folded feed line was introduced, as seen in Figure 19, to make room for the differential feeding scheme and to match the impedance. High isolation and a consistent radiation pattern could both be attained by using differential feeding technology. According to the measurement data, the impedance bandwidths for the two polarizations (VSWR 1.5) were 19.3% (3.14–3.81 GHz) and 20.3% (3.10–3.80 GHz), respectively. Over the whole operating band, the suggested antenna had a high isolation of over 43 dB. Within the operating frequencies, the measured gain was more remarkable than 8.1 dBi [82,83,84,85].

Two rectangular patches and a connected feeding arrangement were combined to create a small BS antenna. The antenna dimensions were 80 mm × 75 mm × 19 mm. The suggested antenna covered the 5G n78-band with a broad bandwidth of 3.15–3.67 GHz for the VSWR < 1.50. Within the whole operating band, the half power beam width (HPBW) was roughly 67°, the FBR was above 20 dB, and the maximal gain was approximately 8.20 dBi [86].

A cross dipole antenna for dual-band sub-6 GHz 5G applications, 8-parasitic patches, 8-radiating patches, and a reflector made up the majority of the suggested antenna presented. The polarization characteristic of the proposed antenna was ±45°. The overlapping bandwidth range was 2.77–5.31 GHz, and the gain fluctuated between 7.60 and 8.40 dBi. With higher operating frequencies, the gain rose. The port isolation was greater than 26 dB, while the cross-polarization level was less than −28 dB [54]. A brand new dual-polarized base station antenna element has been suggested for LTE, 3G, and 5G mobile communication systems where the structure consists of two orthogonal diamond dipoles, one of which has two circular slots carved into it, while the other is a real diamond. A cross pair of two dipoles could produce polarized radiation at a ±45° angle. Every one of the dipoles had two pairs of branches in its diamond patch. Due to its high cross-polarization discrimination (>13 dB), consistent gains of roughly 8.5 dBi in 1.40–2.7 GHz and 5 dBi in 3.80–4.2 GHz, and high front-to-back ratio (>22 dB), the dual-polarized antenna element of the base station has enough bandwidth and very good radiation properties. The designed aspect of the antenna is commonly utilized in the BS antenna array because of its straightforward structure and outstanding performance [87]. A dual-polarized loop-shaped dipole antenna was designed where the suggested antenna had a small enough structure to be used in a 5G BS. It comprised two feeding baluns and a radiator shaped like a cross. To further reduce the size, the antenna’s radiating arms were folded downward. The simulation results demonstrated that the suggested antenna’s radiation pattern is stable. In the operating bandwidth of 3.68 to 4.05 GHz, the peak gain was greater than 6.7 dBi. The antenna is only 0.145λ_0_ in height, while the radiator is roughly a quarter wavelength in size [88].

**Figure 20:**
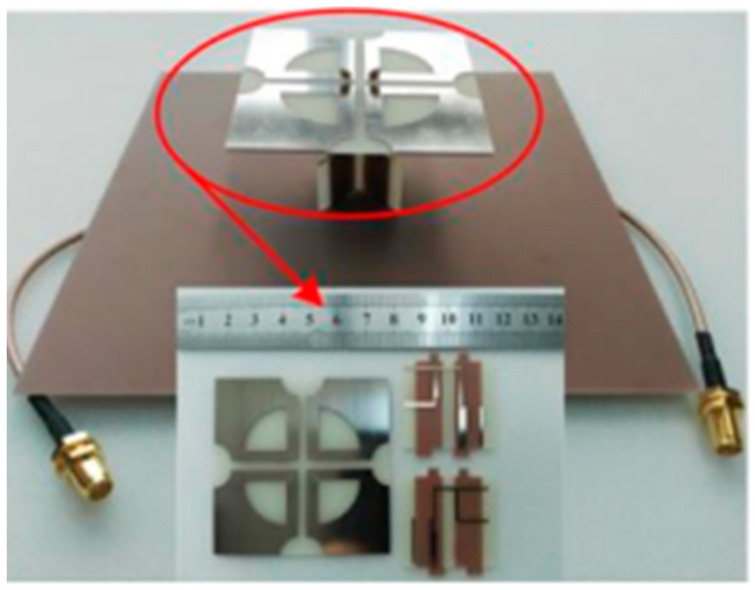
Dual-polarized cross-dipole antenna with fan-shaped slots and Γ-shaped feeders on the front and rectangular patches on the back [89].

For BS applications, a dual-polarized broadband antenna with interference-canceling features is offered. The three parts of the suggested antenna are the feed structure, the main radiator, and the reflector. To achieve ±45° polarization, the central radiator, for instance, contains two crossed dipoles. Between the primary radiator and the feed’s reflectors are two vertical boards with Γ-shaped feeders on the front and rectangular patches on the back. A reflector was used underneath the antenna to produce high gain and unidirectional radiation. A C-shaped stub was further positioned next to the lead to filter undesirable frequency ranges. A prototype antenna was built and tested, as illustrated in Figure 20. The findings from the measurements showed that a notched band with a 52.6% bandwidth existed between 2.27 and 2.53 GHz (VSWR 1.5). The HPBW was roughly 60 degrees, the observed isolation was over 25.4 dB, and the overall operating band average gain was 7.57 dBi. The suggested antenna has the following benefits: small size, dual-polarization operation, band suppression properties, excellent impedance matching, sturdy structure, perfect PCB design, and straightforward process, making it appropriate for next-generation wireless and making it possible for it to be widely utilized in communication systems [89,90].

The new dual-loop array antenna has four rectangular loops and four trapezoidal loops printed on the front and back of the substrate. These loops are each positioned over a flat square reflector and are intended for use in current and future applications involving base stations. In order to achieve polarization states of less than 45 degrees, all eight loop radiators were energized at the same time by a feed network that had been thoughtfully designed. The behavior of a trapezoidal loop is analogous to that of a folded (electric) dipole. Magnetic dipoles are the primary effect that rectangular loops possess. The combination of these two loop arrays resulted in a magnetoelectric (ME) loop antenna that demonstrated a consistent directional pattern with high cross-polarization discrimination (XPD) values over an operating fractional bandwidth of 45.5% from 1.7 to 2.7 GHz. This magnetoelectric (ME) loop antenna covered the frequency range from 1.7 to 2.7 GHz and it was possible to acquire it. The results of the simulation were confirmed by the fabrication and measurement of a prototype, which showed that the horizontal full width at half maximum (HPBW) varied between 63° and 70° and that the XPD values were greater than 20 dB on the central axis and greater than 10 dB on the axis. The total angular range covered by cellular service was −60° ≤ θ ≤ 60° [91].

The main radiator, feeding balun, reflector, and two parasitic loops are the four components that comprise the dual-polarization filter wideband dipole-antenna for base station application. This antenna has a compact size of 50 mm × 50 mm × 31.8 mm. In order to achieve adequate filter performance while simultaneously expanding its bandwidth, a dual-polarized dipole antenna requires only two parasitic loops instead of more complicated filter circuits. Consequently, two distinct radiation zeros are produced, each of which is controlled independently by two parasitic loops. Simple stubs with open ends can be added to the arms of the dipole so that the selectivity of the upper stopband can be improved even further, as can the bandwidth of the band.

Therefore, the bandwidth can be changed from 7.4% to 47.6%, and the gain that can be accomplished can range from 8.6 dBi at 2.7 GHz (in-band) to −10 dBi at 2.9 GHz (out-of-band). For the demonstration, a wideband dipole antenna with dual polarization was implemented. According to the findings of the measurements, the proposed antenna was 48.7% (1.66–2) [92].

A radiating and feeding patch created a new low-profile dual-polarized patch antenna for 5G base stations. The radiation field is excited into dual polarization when a coupled feed field is present. In order to achieve more accurate impedance matching, the edges and corners of these two patches are routinely cut. The processing cost is reduced, and the structure is greatly simplified because only one substrate stands between the two patches. The patch antenna is only 8.7 mm in thickness and measures 32 mm on each side. Its operating frequency range is from 3.3 to 3.7 GHz. In the operating band, the connection isolation coefficients |S11| and |S22| are less than 15 dB, the connection isolation coefficients |S21| are less than 19 dB, and the cross polarization discrimination (XPD) is greater than 27 dB. In addition, a (1 × 3) antenna array was designed using this antenna element as the basis for the design. The antenna array could attain the reflection coefficients of |S11| and |S22|. In the frequency range of 3.3 to 3.7 GHz, the S11 return loss was below −10 dB and port isolation was less than −20 dB. The radiation pattern was both symmetrical and stable, and the H-plane had a half width that ranged from 65 degrees to 76 degrees. The use of this antenna in 5G massive multiple-input multiple-output (MIMO) applications has been evaluated and found to have promising potential [93].

#### 3.1.2. 2D-Model Antennas

**Figure 21:**
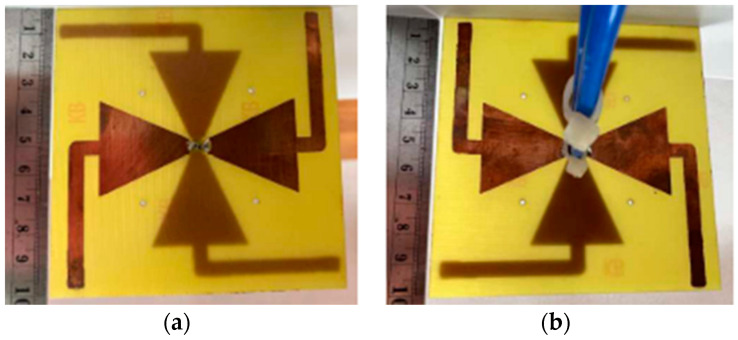
Dual-band, dual-polarization antenna with a pair of bowtie and bent strip-line cross dipoles: (**a**) front, (**b**) back [87].

A dual-band, dual-polarization BS antenna was designed for 5G mobile communication. This was made up of two bowtie-shaped cross dipoles that were bent to create two linear polarizations (±45°), as seen in Figure 21. The dual operating frequency values of 700 MHz and 1800 MHz were provided by the bowtie and bent strip-line dipole. At 775 MHz and 1850 MHz, the antenna exhibited an impedance property of approximately 15.80% and 12.0% for S22 ≤ −10 dB (−45° polarization), and S11 less than or equal to −10 dB (+45° polarization), respectively. In addition, the operational band was given the isolation between the two polarizations, with |S12| being approximately −33 dB and −30 dB at 775 MHz and 1850 MHz, respectively. At 775 MHz and 1850 MHz, the antenna’s measured peak gain was between 1.26 and 4.76 dBi. Additionally, dual-polarization over the two operating bands as well as the omni-directional radiation patterns were obtained. The antenna was designed proportionally for BS applications for 5G mobile communication [87].

For 5G WLAN applications, a rectangular microstrip patch antenna with an H-shaped slot operating at 4.8 GHz is suggested. The H-shaped slot has been utilized in a rectangular patch of the microstrip patch antenna in order to improve the VSWR, gain, reflection coefficient, and bandwidth of the antenna. The suggested antenna was designed in order to meet the requirements of the 5G WLAN applications. The thin planner profile of the microstrip patch antenna is helping it become more popular as they are easily mountable on the planner surface of space-born applications such as aircraft and missiles. For the frequency of 4.8 GHz, the reflection coefficient and VSWR for this antenna design were −25.44 dB and 1.11, respectively. This work developed the suggested antenna with microstrip line feeding [94].

Using a slot feedline and a touch-coupled feeding scheme, a lightweight slotted rectangular microstrip patch antenna for use in 5G wireless applications was proposed, would have high gain and minimal cross-polarization characteristics. The model that has been suggested operates in the sub-6 GHz band and has a frequency span of 3.5 GHz in addition to a bandwidth of 206 MHz. The top layer of the low-k PCB has a compact slotted patch that measures 32 by 25.7 mm^2^, and the backside of the second layer has a full ground applied to it. In addition, the middle layer contains several rectangular slots, which are there to improve the impedance bandwidth, gain, and efficiency as well as lower the reflection coefficient. The overall measurements are 43.36 mm by 35 mm by 1.575 mm^3^ in volume. In addition, utilizing the slotted tape that was previously mentioned raised the radiative efficiency to 83% with a gain that could reach up to 6.58 dBi, which improved the overall performance. This particular antenna configuration has a reflection coefficient (S11) of 28.774 dB and a cross-polarization of only 70.6 dBi, which is considered low. The design that was proposed is a strong contender for use in 5G massive MIMO applications [95,96].

For 5G applications, elliptical microstrip patch antennas with and without slots operating at 3.5 GHz have been designed, and their performance examined. For the 3.5 GHz wireless 5G spectrum, three potential designs of small, affordable, wide-band microstrip patch antennas employing FR4 substrate were examined. The suggested antennas have bandwidth values of 1.13363 GHz, 1.0265 GHz, and 2.1562 GHz at −10 dB return loss with −41.31 dB, −43.95 dB, and 20.44 dB, respectively. The resulting gain was 4.45, 4.36, and 4.42 dBi, whereas the obtained directivity was 4.577, 4.906, and 5.0 dBi. The proposed antennas also appear to have the potential to be included in mobile devices [97].

Low-profile multi-slot patch antennas for long-term evolution (LTE) and 5th generation (5G) communication applications consist of a step patch and a ground plane. Three slots were inserted into the patch to achieve the required operating bandwidth. The insertion slot enhanced the capacity effect, whereas the prototype antenna covered the operating frequency band (S11 ≤−10 dB) ranging from 3.15 to 5.55 GHz, supporting (N77/N78/N79) for the 5G wireless communication sub-6 GHz and LTE (22/42/43/46) bands. The broadband antenna offers an omni-directional and stable radiation pattern, excellent gain, and compact size, making this design suitable for wireless fidelity (Wi-Fi), wireless local area networks (WLAN), LTE, and sub-6 GHz 5G communication applications [98].

A T-slot on a rectangular microstrip patch with defective ground structure (DGS) makes up a slotted ground plane microstrip patch antenna for 5G wireless communications below 6 GHz. The suggested antenna’s ground plane is a modified C-shaped slot. To further enhance the performance of the antenna, the modified C-slot has specific cuts on the bottom and top of the modified C-shape. The suggested antenna’s gain is increased by integrating a reflector into the design to focus the side lobes and strengthen the main lobe of the radiated signal. The presented antenna uses insertion feeding and is constructed on a FR-4 epoxy substrate. The antenna has a size of 28.03 mm × 23.45 mm × 5.35 mm and dimensions of 5.49 dB in gain and 7.12 dB in the directivity at their maximums. The bandwidth of the suggested antenna was between 4.775 GHz and 5.049 GHz [99]. With dimensions of 35 mm × 40 mm × 1.6 mm and a dielectric constant of 2.55, the compact 5G sub-6 GHz wireless patch antenna was placed on a Taconic TLX-8 substrate. The antenna’s peak gain was 6.83 dBi, and its frequency of oscillation was 5.6 GHz. With gains of 6.97 dBi and 5.97 dBi, respectively, the addition of a parasitic ring resonator close to the feed-line resulted in a double resonance at 5.6 GHz and 6.6 GHz. Through inserting a ring-shaped resonator in the ground, antenna miniaturization is also possible. As a result, the resonant frequency changed from 5.6 GHz to 3.8 GHz [100].

### 3.2. Sub-Arrays

High gain, a stable radiation pattern, and a wider frequency band are three of the most critical requirements for 5G antennas. Due to the fact that a single-element antenna cannot fulfill these requirements, a multi-element array antenna has been designed to fulfill them. The following is a discussion of the two types of sub-array configurations, both of which were considered by the MIMO technique (multi-ports).

#### 3.2.1. Symmetric Array

**Figure 22:**
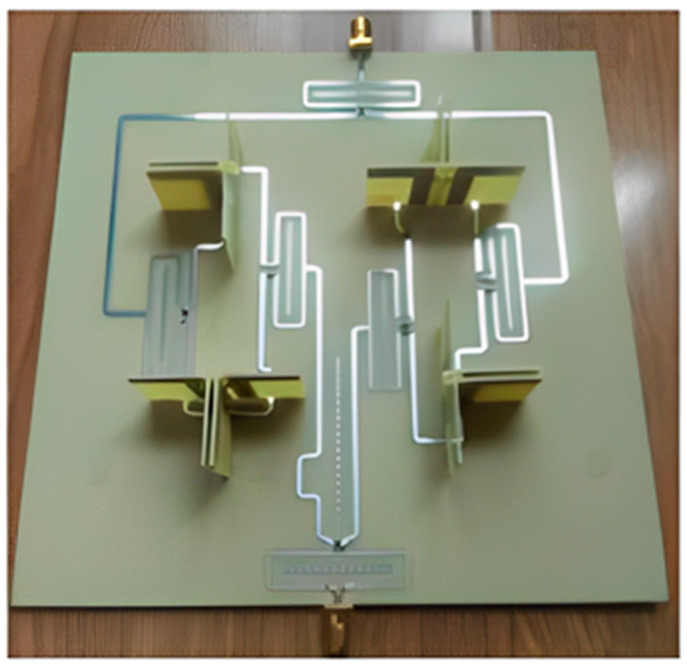
3D of a dual-polarized dipole with halved volume (4 × 4) sub-MIMO array antenna [101].

A dual-polarized antenna that occupied one-half the volume of the traditional crossed dipole antenna was developed. It serves as the basic array element and comprises two back-to-back folded dipoles (2 × 2 sub-array). For each dipole, a portion of its feeding line and one dipole arm are set along the vertical direction. In contrast, the remainder portion of the feeding line and other dipole arms are formed along the horizontal direction, as shown in Figure 22. The dimensions of the antenna size were 38.4 mm × 19 mm × 21.7 mm. The orthogonal currents on the two arms of the dipole could synthesize the slant currents, thus generating ±45° polarization. The proposed antenna element was designed to work at 3.4 to 3.6 GHz band with VSWR < 1.5. Its longitudinal dimension was only 19 mm at 3.5 GHz, about half of the traditional crossed dipole. Then, a sub-array that adopted four sequentially rotated elements and appropriate phase assignment was investigated. The performance of the proposed array was better than that of the regular array. Therefore, the sub-array can be a good candidate for 5G massive MIMO antenna applications [101].

A 4-port wideband cavity-backed antenna for use in indoor BSs is presented. The antenna consists of an X-shaped isolating block and four feeding monopoles that are orthogonally and symmetrically positioned in the cavity’s aperture. The cavity has a square aperture. The modes contributing to the coupling were found using a unique technique depending on characteristic modes analysis (CMA). This investigation led to the suggestion of an X-shaped isolating block put in the cavity’s center to improve the port isolation. With measured impedance bandwidth (S11 < −10 dB) spanning from 1.55 GHz to 6 GHz (118%), a wide-band 4-port antenna with unidirectional radiation patterns was created, covering the majority of sub-6 GHz 5G bands. With 16 dB of minimal measured isolation between ports and an efficiency of more than 84%, the suggested antenna offers four independent radiation patterns. A (4 × 4) MIMO simulated system with an ECC of less than 0.5 was used to demonstrate the compatibility of MIMO in various propagation scenarios. The antenna, which has dimensions of 129.5 mm × 129.5 mm × 28.2 mm and operates at a frequency of 1.55 GHz, is simple to construct. The antenna also has the benefit of avoiding complicated feeding mechanisms with directional couplers or baluns [102].

For 5G wireless communication applications, a brand new dual-polarized dual-band shared-aperture antenna array has been suggested. A LB antenna and four high-band antennas were combined to form this new antenna array. An antenna operating in the low band (0.68–0.99 GHz) and four 5G MIMO antennas operating in the high band (from 3.3 to 5.1 GHz) made up the suggested antenna array. Antennas operating in the low band (from 0.68 to 0.99 GHz) and high band (between 3.30 and 5.10 GHz) were both loop-shape radiators supplied by the Y-shape feed, which simplified the design model while achieving broadband. Incorporating four HB antenna elements into the LB antenna allowed for the resolution of coplanar, dual-band, and shared-aperture issues. According to the simulations, the suggested coplanar antenna array achieved an impedance bandwidth of 43% in the LB (between 0.68 GHz and 0.99 GHz) and 37% in the high band (3.30–5.1 GHz) [103,104].

A sub-6 GHz uni-planar MIMO antenna system for 5G-capable smartphones was designed. Following the idea of pattern diversity, a MIMO antenna was made up of four symmetric loop-shaped radiators that were positioned at every corner of a mobile phone board. A single-antenna element had an impedance bandwidth of 1.28 GHz (3–4.28 GHz) for S11 ≤ −6 and equaled 720 MHz (3.18–3.9 GHz) for S11 ≤ −10 dB. Its resonance frequency was 3.5 GHz. With an antenna efficiency greater than 90%, a peak gain of 3.64 dBi for a single antenna element was noted. For the MIMO configuration, isolating of >10 dB between the characteristics of the antenna was attained. Additionally, the MIMO antenna design offered sufficient radiation coverage to support several mobile phone board sides, which is a crucial characteristic for upcoming 5G-capable devices [105].

**Figure 23:**
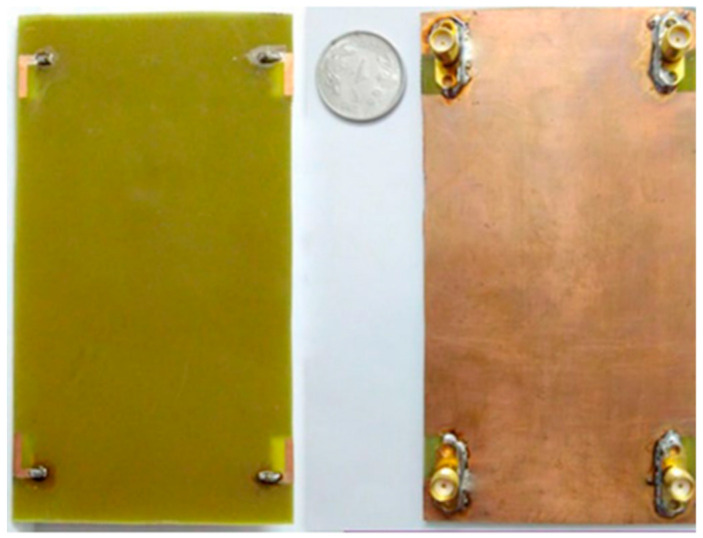
Front and back plane of the fabricated L-shaped and ground plane with four etched slots for the (4 × 4) MIMO antenna [106].

A low-profile planar four-element MIMO antenna for wireless handheld devices has also been proposed. Simple planar L-shaped monopoles in the shape of each unit antenna are mounted over etched non-ground areas measuring 10 mm × 5 mm. The improved antenna elements provided an isolation of more than 18.8 dB without any need for an extra decoupling structure for the three 5G New Radio bands that fall inside the C-band: the n-77 band (3.30–4.20 GHz), n78 band (3.30–3.80 GHz), and n79 band (4.4–5 GHz). As illustrated in Figure 23, the designed antenna was constructed on an FR-4 substrate with dimensions of 120 mm × 65 mm × 1.6 mm. The measured and discussed antenna properties included reflection coefficient, radiation pattern, mutual coupling, and gain. The estimated envelope correlation coefficient was less than 0.018 for the considered frequency range. The suggested MIMO antenna’s simplicity and compactness leave enough room inside the handheld mobile terminal to integrate other circuits. Investigation into the integration with lower generation antennas was further conducted, and the findings show that mounting the suggested antenna system on both of the long arms of the ground plane provides enough room for the integration of a lower generation antenna without negatively impacting the performance of either [106,107].

#### 3.2.2. Non-Symmetric Array

A non-symmetric array was considered as a (1 × n) sub-array in the arrangement. A wideband dual polarization antenna was suggested for 4G/5G communications applications. The antenna element was made up of two open-loop, bipolar dipoles with three different resonant modes pushed closer to one another to create a wide bandwidth. An extra “U”-shaped slot that was etched around the feed point made the antenna element’s input impedance equal to 50 ohms. The constructed antenna element had high port-to-port isolation (higher than 25 dB), a 75.90% impedance bandwidth (VSWR ≤ 2), and operated in the 1.8 to 4.0 GHz frequency range. It also offered a beamwidth of 67° ± 1° in the H plane and 68.7° ± 3.3° in the V plane, with a gain of 8.5 ± 1 dBi in the supported bands. A 6-element (1 × 6) dual-polarized array with an electrically down-tilt was also created and measured; this array is preferred for communication applications because it achieves a peak gain of 16.8 dBi, a beam width in the H-plane that was comparable to one antenna element, and an electrically down tilt in the V-plane that ranged between 0° and 12°. Six antenna elements were used to obtain such results, and an RF phase shifting module was created. In the near future, this antenna array might be utilized for future 5G communications as well as other applications [108,109].

An innovative linearly dual-polarized antenna array with triple elements (1 × 3) has been suggested for beam width reconfigurable base station communications. A single, dual-polarized antenna unit has the ability to obtain a wide bandwidth of 78.40% for SWR ≤ 2, which ranges between (1.63 GHz and 3.73 GHz) for 2G/3G/LTE and 3.5 GHz of C-band (3.40–3.60 GHz) applications if arc-shaped corners on the radiated patches are removed. This can be conducted by cutting off the corners of the radiated patches. Additionally, because of the box-shaped reflector and intrinsic performance of the ME dipole antenna, the dual-polarized antenna element has an approximate gain of 11.5 dBi on average. In the final result, a 3-element antenna array with a widely adjustable beam width in the E-plane as well as the H-plane was investigated. This antenna array could meet the needs of smart applications in the future and was made with a two-stage circuit and numerous power dividers [110].

Four arrays with more than 60 dB isolation in the 5.15–5.925 GHz band were used to illustrate the process of designing a dual-polarized linear antenna array with enhanced port-to-port isolation. The single antenna adopts a dual-polarized electromagnetic coupling microstrip antenna, and the isolation between ports did not exceed 25 dB. Array isolation can be significantly improved by using a dedicated feed network. A mathematical model was established based on theoretical analysis to describe the isolation between ports. Circuit and full-wave simulations were performed to show the effect of electromagnetic coupling between the antennas and/or microstrip lines in the feed network and the effect of the phase shifter/power divider selection on isolation. The manufactured prototype featured a gain of approximately 14 dBi, a minimum polarization purity of −27 dB within the main lobe, and was close to the expected isolation of >57 dB over the entire operating range [111].

A compact, highly isolated MIMO antenna system for wireless applications in 5G-connected devices has been considered with the size of 92 mm × 88 mm and consisting of two elliptical antennas symmetrically arranged next to one another. To provide the various MIMO antennas proposed, two decoupling methods were applied: neutralization and DGS. The single and MIMO antennas were modeled and analyzed, then built and measured. A good agreement was obtained between the measurements and simulations. These configurations were designed to cover the frequency range of 3.4 GHz to 3.8 GHz and showed a highly satisfactory performance exceeding −30 dB, while reducing the mutual coupling between the antennas that make up the system. The MIMO parameters of diversity such as ECC, diversity gain (DG), and overall performance were also investigated for every one of the proposed MIMO systems. Therefore, the results showed that the proposed two antenna configurations are quite suitable for 5G MIMO applications [112].

The triple band MIMO antenna for the 5G mobile terminal applications consists of 4-ports/2-resonators, each with a ground plane that is (50 mm × 50 mm) in size and two concentric circular slot ring radiators engraved into it. A 50 Ω microstrip line perpendicular feeds the antenna to the top layer. Decoupling methods have been utilized in order to suppress mutual coupling between two resonators. The vertical arrangement of leads and terminals reduces the mutual coupling between the two terminals and increases isolation. The antennas operate in multiple frequency bands (3.35–3.69 GHz, 24–28 GHz, and 37–40 GHz) and frequency ranges, with a focus on 3.5 GHz, 26 GHz, and 38 GHz-like assignments for 5G. The antenna provided a 2.7–7.8 dB gain and 0.49–0.85 radiation efficiency in the operating frequency band and the diversity performance in terms of ECC, diversity gain (DG), and TARC were checked. These were found to be <0.01, >9.99 dB, and less than −10 dB, respectively. The suggested antenna has excellent S-parameters, a low ECC, a decent VSWR, a TARC, a good radiation pattern, and a high gain. The production and testing of antennas. Results from the simulation and measurements were in good agreement. Applications for 5G mobile terminals and smart wearables have a lot of potential [113]. A novel dual-polarized, printed-dipole antenna design was proposed for base station antennas for 5G mobile communication systems operating in the 3.30–5.90 GHz band. Each crossed dipole was excited by a micro-tip wire stub fed from a standard 50 Ω of coaxial cable. A configuration of synthetic dipole arms using third-order Bezier curves. This method provides precise tuning of the mutual coupling of the crossed dipoles and extension of the antenna’s operating frequency band. The significant improvement in antenna performance compared to known prototypes was achieved after numerical full-wave optimization of the dipole profile. The characteristics of the proposed 8-element antenna array with improved planar dipoles were investigated. In the 3.30–5.90 GHz (57%) frequency band, the reflection coefficient was −15 dB, the isolation was −28, −30 dB, and the beam width of the −10 dB level corresponded to 120°−4°/+10° [114].

4G/5G and combined (sub-6 GHz/mm-Wave) multi-band mMIMO can assist in solving more problems of 5G system structure such as bandwidth shortage and complex hardware requirements. The quality of the 5G antenna performance depends on the parameter chosen, the results of antenna work such as gain, power consumption, efficiency, bandwidth, and the surrounding environments of the antennas for the base station and smartphones. For instance, enhancement in the impedance bandwidth and gain in mMIMO will provide the maximum coverage area for the base station and smartphones. High channel capacity will offer high throughput and spectral efficiency. The diversity of pattern characteristics (gain, polarization) makes massive MIMO a good choice for 5G applications.

Table 5 summarizes the types of 5G antennas (single and sub-array elements) as candidates for massive MIMO applications.

These models are used depending on which is the most suitable for design in base station or smartphone applications. Many smartphone techniques use 2D model single and symmetric subarray elements. The size and requirements of the smartphone to design it do not allow for the use of 3D models or non-symmetric subarrays in contrast to the base station, which can be used in one or many designs. Base station technique requirements permit the use of these types of 5G antennas in 2D and 3D models, and symmetric or non-symmetric sub-arrays for small and large sizes.

## 4. Challenges and Future Directions

Massive MIMO is clearly superior to conventional multiple antenna systems. Massive MIMO and the most recent 5G technology could work wonders for wireless networking. However, a number of problems continue to prevent mMIMO from being used practically [115]. Several issues for hardware components such as choosing the material, size, cost, and characteristic features (bandwidth, gain, efficiency, mutual coupling, etc.) can be faced for both types of applications.

The almost unlimited variety of devices will cause various design issues, which in turn will be exacerbated by the variety of frequencies in 5G. To support the devices operating on diverse spectral bands, the spectrum must be flexible [116]. The sub-6 GHz frequency band is becoming the band of interest for 5G communication to solve these issues. In 5G sub-6 GHz, base station techniques use single and multiband designs through different frequency ranges, which still face some challenges. With multi shapes for single and arrays such as ME dipole-cross patches, patch sub-array, multimode-slotted structures, and other designs, high gain and efficiency can be realized. For smartphone designs with LTE (42/43/46 and 47) frequency bands, the inverted-F stub fed hybrid-loop antenna, T-shaped coupled-fed slot antenna, and planar inverted F-antenna (PIFA) can be used with high performance.

Frequencies do not present a wide variety of challenges as the current questions are where to put the antenna, how many are needed, and how to keep them from interfering with each other, especially after the recent demand for a 5G network [117]. In the design of a symmetrical and asymmetrical array, the placement of the antennas is arranged with a specified number of elements. Symmetrical and non-symmetrical planar rectangular arrays such as (4 × 4) and (4 × 1) models of elements, as an example of base station techniques, arranged the antennas and studied the effect of the single element and arrays on the radiation pattern directivity, gain, and efficiency. The arrangement of antennas in smartphone design is to put them at the edges as symmetrical or non-symmetrical for two sides or one side such as the (8 × 8) model of elements, with each four arranged at each edge. These can obtain high results with the specified dimensions between elements and perfect isolation.

The effects of mutual coupling due to the close spacing of the MIMO antenna elements and their impact on the correlation, which ECC determines, are challenging [118]. However, the compact size and decoupling techniques for the enhancement of isolation between the antennas has relatively resolved the problem, and the effect on the characteristics’ results was positive. The geometry of the antennas was installed in a compact size, which could contribute to the low mutual coupling and high spectral efficiency. It can be noted that the 2D and 3D placement of antenna elements have become the support for mMIMO base stations. Nevertheless, implementing antenna arrays in 2D or 3D can dramatically decrease energy efficiency and enhance coupling effects. In order to dramatically improve the spectral efficiency, it is possible to increase the spacing of the array element by using decoupling techniques. The 3D-model element rectangular planar lattice array uses three decoupling methods: a ferrite chock ring, a rectangular ring resonator, and a unique baffle design [28]. A 3D array massive MIMO antenna with a compact size can be used with decoupling and without decoupling methods such as cylindrical, triangular, and hexagonal models for enhancement results. The size of the smartphone and the requirements for design in the massive technique need decoupling methods, which remains one of the key features of 5G MIMO antennas. Thus, the slot method affects the decoupling component positively to enhance isolation between other elements [58]. Spatial diversity and polarization techniques are used with a mMIMO switchable frame antenna array as a decoupling technique to reduce the coupling effects and enhance isolation [63]. Inverted-F, T-shaped decoupling stub, self-decoupling, defective ground structure, and other techniques are also used as decoupling methods.

As a future direction, to face the challenges of the hardware, the characteristics of the components, and their modification and enhancement, a metamaterial technique with unique properties will used as isolation, which is attributed effectively to the enhancement in the size, gain, efficiency, bandwidth, and other aspects.

In smartphones, maintaining the SAR within the predetermined limits is difficult since MIMO systems have an increasing number of antennas [119]. Several techniques in design antennas are used to reduce the SAR results to <2, which is very safe for user applications. Furthermore, increasing the bandwidth is a much more important goal to decrease the SAR and high efficiency to ensure safe user handling of the mobile device.

Low-cost hardware is considered as the upcoming demand for 5G systems since it requires an economy of scale in manufacturing. Despite the fact that the BS has numerous antennas deployed, a high array gain can still be attained due to hardware shortcomings, which might result in channel estimate error and a capacity ceiling. The user side will experience severe hardware impairments compared to the BS side [120]. The high cost of mMIMO in smartphones also faces a challenge that affects the price of the devices on the market. Therefore, a reasonable cost and effective technical equipment can achieve perfect results.

Finally, the selection type of the application is relative to the environment surrounding the application and the ways to use it. The environment must provide the optimal propagation conditions for mMIMO systems to function well. As a future direction, a three-dimensional massive MIMO array of the base station for maximum coverage and low SAR for a smartphone with a flexible spectrum band ensures good implementation for mixture (5G/6G) and 6G networks, whereas a metamaterial is a good choice to improve the overall performance [121].

## 5. Conclusions

Massive MIMO is a crucial part of the 5G infrastructure. 5G massive MIMO uses a large number of antennas for both base stations and mobile devices. Increasing the number of antennas at the base station in relation to the number of users and smartphones enhances the performance of a massive MIMO system. Both the mutual coupling and spatial correlation between antennas play a role. For massive MIMO to work in either scenario, 5G antennas will need to have exceptional characteristics. As a result, many 5G antennas meet the criteria for the expansion characteristics of massive MIMO systems applicable to both methods. For sub-6 GHz frequencies, massive MIMO antenna systems in 5G mid-band base stations have been extended to support a maximum of 256 antenna elements, while for smartphones, the maximum is 20.

## Figures and Tables

**Table 1:** Massive MIMO techniques: enhancement and support for base station applications.

Ref.	Antenna Schemes	Topology	MIMO Model	Supported Massive Tech.	Enhancements				
					Compact	B.W	Isolation	Gain	Eff.
[28]	UB-FSS-LB	Rectangular	4 × 4	Yes	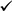	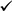	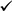		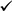
[30]	ME dipole-cross patches-dotted slots	Rectangular	4 × 4	Yes	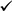	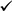			
[32]	Bowtie dipole aerial	Rectangular	4 × 4	Yes	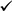	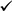	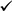		
[34]	Patch sub-array	Rectangular	8 × 8	Yes	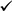		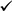	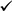	
[35]	Multimode-slotted structure	Rectangular	11 × 11	Yes	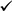	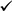	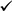		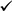
[40]	Patch sub-array	Rectangular, Triangular, Hexagonal	5 × 4	Yes	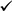			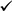	
[41]	Multimode square patch-stacked polyhedron ring	Ortho-hexagonal		Yes	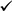		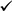	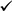	
[43]	Tapered slot antenna (TSA)	Cylindrical		Yes	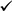	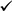	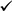		
[50]	Square patch with two merged u-slots-dipole	Rectangular		Yes				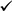	
[55]	Microstrip patch	Rectangular	4 × 4	No	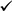	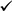		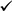	
[56]	Slotted microstrip patch antenna-(NZI-ENG) metamaterial decoupling structure	Cylindrical		No	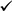		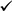		

**Table 2:** Comparison of sub-6 GHz mMIMO antenna techniques.

Ref.	No. of Elements/Ports	Size (mm)	B.W (GHz)	Isolation (dB)	ECC	Gain (dBi)	Eff. (%)
[28]	16/32	344 × 344 × 63	3.3–5.0 (UB) 0.69–0.96 (LB)	>30	< 0.1	7.3 (UB) 8.6 (LB)	>90
[30]	16/32	90 × 90 × 19.5 (single element)	3.25–5.35	-	0.004	-	-
[32]	16/32	200 × 200 × 32	2.8–4.0	27	-	9.1	-
[34]	64/16	333.3 × 333.3 × 1.6	3.45–3.68	25	-	5.4 (one-port)	30.887 (one-port)
[40]	20/5	280.5 × 56.1 × 2 (single sector)	3.36–3.5	12.3 14.2 13.9	-	19.73 (rectangular) 13.45 (triangular) 14.37 (Hexagonal)	-
[41]	288/144	648 × 648 × 258	3.65–3.81	>31	-	16.7 Sub-array (1×4 ant. units)	-
[43]	24/24	280 × 194.4 × 1.6	3.0–4.2	>20	<0.01	-	-
[50]	32/32	440 × 440 × 1.6	2.4–2.62 3.4–3.6	-	-	6 (Single Patch)	-

UB: upper band; LB: lower band.

**Table 3:** Massive MIMO technique enhancement and supposition for smartphone applications.

Ref.	Antenna Schemes	MIMO Model	Supported Massive Tech.	Enhancements				
				Compact	B.W	Isolation	Gain	Eff.
[58]	Slot antenna-open ended decoupling slots	18 × 18	Yes	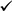	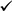	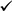	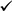	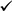
[59]	Inverted π-, longer and shorter inverted L-shaped, open slot, antennas	8 × 8 (LB) 6 × 6 (HB)	Yes	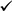	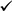			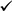
[60]	Inverted-F stub fed -hybrid loop antenna	10 × 10	Yes	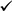	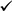	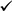		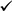
[61]	T-shaped coupled-fed slot antenna	10 × 10	Yes	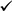	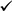	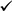		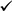
[62]	Planar inverted F-antenna (PIFA)	8 × 8	Yes	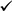	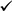		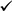	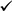
[63]	Switchable frame antenna	8 × 8 2 × 2 (LB) 6 × 6 (HB)	Yes	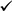	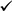	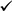		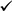
[64]	Diamond-ring slot antenna		Yes	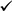	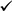	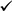	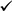	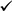
[65]	Planar inverted-F antennas (PIFAS)	18 × 18 16 × 16 14 × 14 12 × 12 10 × 10 8 × 8	No	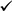	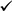			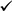
[69]	Two open-end slots—QMSIW antenna	12 × 12	No	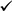		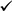		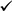
[71]	Inverted-F antennas (IFAs)	10 × 10	No	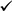	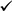	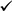		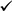
[74]	Inverted L-shaped monopole		No	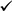		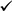	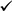	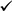
[76]	Balanced open slot antenna	8 × 8	No	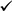		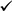		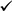
[77]	Orthogonal-mode dual-antenna pairs-shared radiator	8 × 8	No	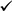	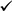	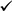		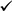

LB: lower band; HB: higher band.

**Table 4:** Comparison of the previous references for massive MIMO for smartphone applications.

Ref.	No. of Elements/Ports	Size (mm)	B.W (GHz)	Isolation (dB)	ECC	Gain (dBi)	Eff. (%)	PLL (bps/Hz)
[58]	18/18	150 × 80 × 1.6	(42/43) 3.4–3.8	>20	<0.01	>5.3	87–93	81
[59]	12/12	150 × 80 × 0.8	(42/43/46) 3.4–3.8 5.15–5.925	>12	<0.15 0.1	-	41–82 47–79	37 29.5
[60]	10/10	120 × 70 × 1.52	(42/43/46) 3.4–3.8 5.15–5.925	≥16 ≥15	≤0.21 ≤0.15	-	82–95 78–96	52.5–53.4 52.8–53.9
[61]	10/10	150 × 80 × 0.8	(42/43/46) 3.4–3.8 5.15–5.925	>11	0.15 0.05	-	42–65 62–82	48 51.4
[62]	8/8	150×75×1.6	(42/43/47) 2.5–2.7 3.4–3.8 5.6–6	>10	<0.01	3–4.5	40–80	-
[63]	8/8	150 × 75 × 0.8	2.49–2.69 3.38–3.6	>15.2	<0.15	N.A	<68	<37

PLL: peak channel capacity.

**Table 5:** Comparison between the reference works.

Ref.	No. of Elements/ Ports	Type of 5G Antenna	Size (mm)	B.W (GHz)	Return Loss (S11/dB)	Isolation (dB)	Gain (dBi)	Eff. (%)
[82]	1/2	3D-model	82 × 82 × 11.8	3.14–3.81	-	>43	>8.1	83
[86]	1/1	3D-model	75 × 80 × 19	3.15–3.67	33	-	8.2	-
[95]	1/1	2D-model	43.36 × 35 × 1.575	3.392–3.598	28.774	-	6.58	83
[97]	1/1	2D-model	55 × 40 × 1.6 44 × 30 × 1.6 46 × 26 × 1.6	3–4.136 3.14–4.167 3.154–5.31	41.31 43.95 20.44	-	4.45 4.36 4.42	>88
[102]	4/4	Symmetric sub-array	129.5 × 129.5 × 28.2	1.55–6	-	16	-	84
[106]	4/4	Symmetric sub-array	120 × 65 × 1.6	3.3–5	-	18.8	4.71	-
[110]	3/6	Non-symmetric sub-array	160 × 16 × 35 (Single ant.)	1.63 to 3.73 3.4–3.6	-	25	12.9 17.3	-
[114]	8/16	Non-symmetric sub-array	95 × 455 × 11	3.3–5.9	15	28–30	-	-

## Data Availability

Not applicable.
